# A persistent invasive phenotype in post-hypoxic tumor cells is revealed by fate mapping and computational modeling

**DOI:** 10.1016/j.isci.2021.102935

**Published:** 2021-08-04

**Authors:** Heber L. Rocha, Inês Godet, Furkan Kurtoglu, John Metzcar, Kali Konstantinopoulos, Soumitra Bhoyar, Daniele M. Gilkes, Paul Macklin

**Affiliations:** 1 Department of Intelligent Systems Engineering, Indiana University, Bloomington, IN 47408, USA; 2 Department of Oncology, The Sidney Kimmel Comprehensive Cancer Center, The Johns Hopkins University School of Medicine, Baltimore, MD 21231, USA; 3 Department of Chemical and Biomolecular Engineering, The Johns Hopkins University, Baltimore, MD 21218, USA; 4 Department of Informatics, Indiana University, Bloomington, IN 47408, USA; 5 Cellular and Molecular Medicine Program, The Johns Hopkins University School of Medicine, Baltimore, MD 21231, USA

**Keywords:** Cell biology, Systems biology, Cancer systems biology, Cancer

## Abstract

Hypoxia is a critical factor in solid tumors that has been associated with cancer progression and aggressiveness. We recently developed a hypoxia fate mapping system to trace post-hypoxic cells within a tumor for the first time. This approach uses an oxygen-dependent fluorescent switch and allowed us to measure key biological features such as oxygen distribution, cell proliferation, and migration. We developed a computational model to investigate the motility and phenotypic persistence of hypoxic and post-hypoxic cells during tumor progression. The cellular behavior was defined by phenotypic persistence time, cell movement bias, and the fraction of cells that respond to an enhanced migratory stimulus. This work combined advanced cell tracking and imaging techniques with mathematical modeling, to reveal that a persistent invasive migratory phenotype that develops under hypoxia is required for cellular escape into the surrounding tissue, promoting the formation of invasive structures (“plumes”) that expand toward the oxygenated tumor regions.

## Highlights

• A fluorescent fate mapping system allows tracking of hypoxic and post-hypoxic cells
• Computational modeling predicts the formation of post-hypoxic invasive plumes
• Simulations show post-hypoxic cells must maintain persistant migration to form plumes
• Tracking cells exposed to intratumoral hypoxia confirms persistent migration

## Graphical abstract

**Figure:**
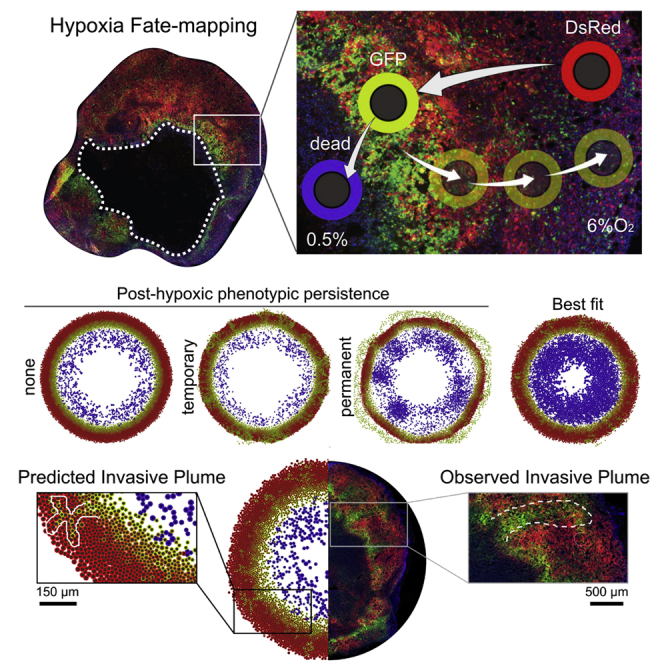

## Introduction

Intratumoral hypoxia, or oxygen (O_2_) deprivation, is associated with an increased risk of metastasis, treatment failure, and worse patient outcome (Gilkes and Semenza, 2013; Muz et al., 2015). Hypoxia occurs in 90% of solid tumors as a result of rapid cancer cell proliferation and inefficient vasculature (Cosse and Michiels, 2008). Studies using an Eppendorf electrode demonstrated that the median partial pressure of oxygen (pO_2_) in solid tumors in patients with breast cancer was 10 mmHg (1.3% O_2_) compared with normal breast tissue whose median pO_2_ was 65 mmHg (8.6% O_2_) (Vaupel et al., 2007).

The cellular response to hypoxia is driven by the hypoxia-inducible factors 1 and 2 (HIF-1 and HIF-2, respectively). The alpha subunits of HIF-1 and HIF-2 are subject to proteosomal degradation under well-oxygenated conditions. Under hypoxia, HIF-1*α* and HIF-2*α* are stabilized, translocate to the nucleus, and heterodimerize with HIF-1*β* (Berra et al., 2001). HIF-1 and HIF-2 heterodimers transcriptionally regulate gene expression when O_2_ is limited. Hypoxia has been reported to regulate the expression of more than a thousand genes (Ye et al., 2018), the majority of which are induced by HIFs. Current methods to detect hypoxia within a tumor are limited to distinguishing cells that are hypoxic at the time of measurement. Consequently, upon reoxygenation, the hypoxic stimulus is lost, and the ultimate fate of the post-hypoxic cells is unknown. To overcome this obstacle, we recently developed a hypoxia fate mapping system that uses a Loxp-Cre approach to drive a permanent fluorescent switch from DsRed to GFP expression once a cell experiences hypoxia. Using this strategy, our preliminary data demonstrated that cells exposed to hypoxia in the primary tumor are 5 times more efficient at forming lung metastases in mouse models of breast cancer (Godet et al., 2019). Moreover, as we monitored tumor progression in an orthotopic mouse model during a timecourse experiment, we visualized the appearance of GFP+ tumor regions lining the necrotic core of the tumor that eventually progress to larger GFP+ areas. Although these observations were insightful, we could not fully characterize the dynamic cellular changes that occur in response to the O_2_ gradients within the tumor. Intravital imaging would allow us to acquire real-time dynamics in select areas of the tumor; however, it is limited to short time intervals where the animal is immobilized on the microscope stage and only small tumor volumes can be analyzed. Therefore, the goal of our current work is to establish a computational model based on *in vivo* and *ex vivo* data that captures the spatiotemporal dynamics of hypoxic cells at large length scales, and use it to explore potential hypotheses driving post-hypoxic cancer cell behavior.

There is a large body of work on mathematical modeling of cell migration in the context of tumor progression (Anderson et al., 2000; Prahl and Odde, 2018; Rangarajan and Zaman, 2008; Stonko et al., 2015). To date, many mathematical models have included hypoxia when modeling the dynamics of tumor growth (Alarcón et al., 2004; Lima et al., 2014; Macklin et al., 2012; Macnamara et al., 2020; Meaney et al., 2019; Rocha et al., 2018). Macklin et al. (2012) used a model for ductal carcinoma *in situ* where cells exposed to hypoxia could not recover to their nonhypoxic state and immediately became necrotic. In the study by Lima et al. (2014), hypoxic cells released proangiogenic factors such as vascular endotheial growth factor (VEGF) into the tumor microenvironment, which stimulated endothelial cells to promote angiogenesis and partially relieved hypoxia. A model combining hypoxia-activated prodrugs (HAPs) with radiation therapy was developed by Meaney et al. (2019). The migratory advantage of hypoxic cells was modeled by Macnamara et al. (2020). However, modeling both hypoxic and post-hypoxic cells has not been attempted due to the lack of experimental data required to build such a model.

**Figure 1:**
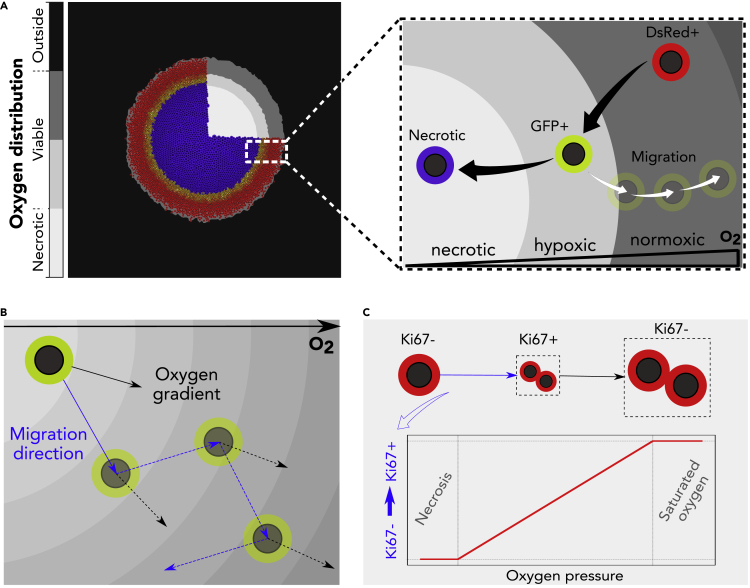
Overview of the computational model (A) The O_2_ distribution in the microenvironment defines the viable and necrotic regions of the tumor (left). Phenotypic changes defined by the concentration of O_2_ and permanent switch to GFP fluorescence (right). (B) Random migration with a directional bias along the O_2_ gradient. (C) Scheme of the cell cycle, where the transition rate from Ki67- (non-cycling) to Ki67+ (cycling) scales with O_2_ availability.

Here, we have the unique opportunity to gather biological data from cells within different tumor regions: necrotic and viable. The tumor cells have distinct phenotypic states according to O_2_ distribution: normoxic cells localize in oxygenated tumor regions (DsRed+), hypoxic cells localize adjacent to the necrotic core (GFP+), post-hypoxic cells localize in oxygenated regions (GFP+) after migration, and nonviable cells localize in the necrotic core (Figure 1A). Experimental data gathered from this setup were used to establish a multiscale continuum-discrete model within the PhysiCell simulation framework (Ghaffarizadeh et al., 2018). *In vivo* O_2_ measurements performed in tumors using an optical probe defined the O_2_ gradient. To obtain motility measurements, we used tumor-isolated cancer cells cultured in an *ex vivo* 3D spheroid model. Using the approximate Bayesian computation (ABC) technique (Beaumont et al., 2002), we calibrated key parameters to model cell motility derived from biological experiments, particularly speed and movement bias (Figure 1B). Proliferation was assessed using Ki67 staining, which marks cells in active phases of the cell cycle (Figure 1C).

To design a representative model of tumor progression, we defined the following parameters to characterize the GFP+ cells: phenotypic persistence time (*T*_*p*_), bias of the cell migration (*b*^∗^), and fraction of cells that can respond to a migration stimulus (*F*_*r*_). Phenotypic persistence time is defined as the persistence of the migratory response once the cell leaves the hypoxic region. The motility bias illustrates the tendency of cell migration to align with the O_2_ gradient. Lastly, the fraction of responders represents the number of GFP+ cells that experience an enhanced migration when compared with the remaining GFP+ population, as suggested by our biological experiments. By testing a range of these parameters, we explored different scenarios predicted by our model and compared them with the *in vivo* observations from tissue sections of the primary tumor both in 2D and 3D. A qualitative comparison with the *in vivo* output demonstrates that our model accurately recapitulates the biological observations of GFP+ invasive structures.

This work combines the hypoxia fate mapping system with computational modeling to gain new biological insights into the dynamical behavior of post-hypoxic cancer cells. The hypoxia fate mapping allows us to visualize invasive tumor structures, while the computational model allows us to explore the cell behavioral hypotheses and dynamics that can and cannot recapitulate the invasive structures. We then “close the loop” using targeted experimental imaging to test the simulation hypotheses. In particular, this integrative approach demonstrates that a persistent post-hypoxic phenotype is needed to explain “plumes” of GFP+ cells in well-oxygenated tumor regions. The work demonstrates how the marriage of imaging and computational modeling can drive advances in understanding hypoxia-driven cancer invasion.

## Results

### Fate mapping intratumoral hypoxia enables characterization of a hypoxic/post-hypoxic phenotype

**Figure 2:**
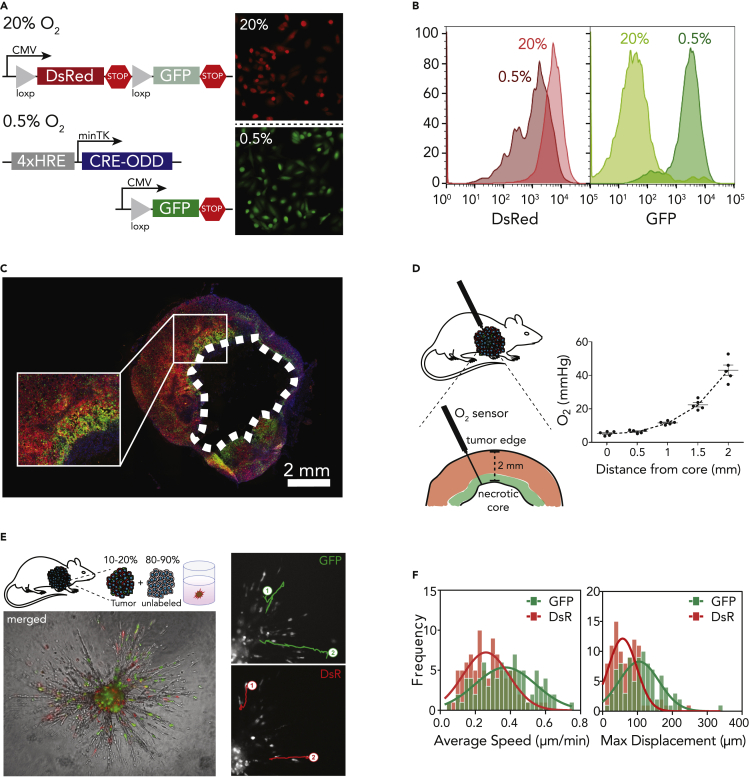
Fate mapping intratumoral hypoxia (A and B) Lentiviral vectors used to generate the hypoxia fate mapping system. Fluorescent images (A) and flow cytometry analysis (B) of the MDA-MB-231 hypoxia fate mapping cells following exposure to 20% or 0.5% O_2_ for 5 days. (C) Fluorescent images of the full cross section of an orthotopic tumor derived from MDA-MB-231 hypoxia fate-mapping cells. (D) O_2_ measurements performed using a fixed-needle microprobe are displayed as a function of distance from the tumor core. Data are represented as mean ± SEM. (E and F) Representative spheroid used to track migrating tumor-derived GFP+ or DsRed+ cells (E) and corresponding average speed and maximum displacement of individual cells (F) fit by Gaussian nonlinear regression.

We recently developed an approach to fate map hypoxic cells *in vivo* during tumor progression in murine models of breast cancer (Godet et al., 2019). The model was developed by delivering two lentiviral vectors to the human metastatic breast cancer cell line, MDA-MB-231. The first vector contains a constitutively active promoter and encodes an expression cassette for a red fluorescent protein (DsRed) flanked by tandem *loxP* sites and followed by a gene encoding a green fluorescent protein (GFP). The second vector contains four hypoxia-responsive elements (HREs) that transcriptionally regulate the expression of a Cre gene which was altered by adding an oxygen-dependent degradation domain (ODD). Consequently, under hypoxia, Cre promotes the cleavage of the DsRed gene leading to the permanent expression of GFP (Figures 2A and 2B).

To investigate the emergence and progression of hypoxia in primary breast tumors, hypoxia fate-mapping MDA-MB-231 cells were implanted in the mammary fat pad of NSG mice. Tumors were harvested, cryosectioned, mounted, and imaged following 15, 20, 35, and 40 days of tumor growth (Figures 2C and S1A). GFP+ cells were localized to perinecrotic regions of the tumor, and GFP+ areas within the tumor increased over time (Godet et al., 2019). We also observed the leading edge of GFP+ tumor areas invading into DsRed+ tumor regions (Figure 2C).

In order to build a mathematical model of our *in vivo* system, we conducted experiments to define key parameters, such as the spatial distribution of O_2_ in MDA-MB-231-derived orthotopic tumors and the proliferative capacity of GFP+ and DsRed+ cells. Using an optical detection probe, O_2_ measurements were recorded at 0.5-mm depth increments within the 2-mm viable rim. The maximum recorded pO_2_ was 43–46 mmHg at the periphery of the tumor, while in the perinecrotic region, the pO_2_ dropped to 6 mmHg (Figure 2D). To assess the proliferative capacity of DsRed+ and GFP+ cells in tumors, we immunofluorescently labeled tumor tissue sections with Ki67, which marks cells in active phases of the cell cycle (Bruno and Darzynkiewicz, 1992). Image analysis revealed that overall, 50 to 55% of cancer cells were Ki67+, which is in line with previously reported Ki67 indexes in MDA-MB-231-derived tumors (Warin et al., 2010). Moreover, there was no significant difference between Ki67 expression in DsRed+ and GFP+ cells (Figures S1B and S1C), suggesting that proliferation is not distinct between the two cell populations.

Given previous findings (Ju et al, 2017, 2020; Lewis et al., 2016) that hypoxia enhances cell migration and invasion, we investigated the migratory-invasive behavior of tumor-derived cells in a spheroid model. In our studies, we isolated GFP+ and DsRed+ cells from primary orthotopic mouse tumors and used them to generate spheroids containing 10–20% tumor-derived cells and 80–90% unlabeled type cells. Using live cell imaging, we tracked individual cells migrating within the spheroid as well as invading into the adjacent collagen matrix (Figure 2E). GFP+ cells displayed a higher mean speed and maximum displacement (*s* = 0.38 *μm*/*min* and *d*_*max*_ = 111.11 *μm*) compared with dsRed+ cells (*s* = 0.28 *μm/min* and *d*_*max*_ = 68.34 *μm*) (Figure 2F). The observations suggest that even after reoxygenation, GFP+ cells are more motile than DsRed+ cells.

### Biological observations calibrate cellular motility in hypoxia computational model

**Figure 3:**
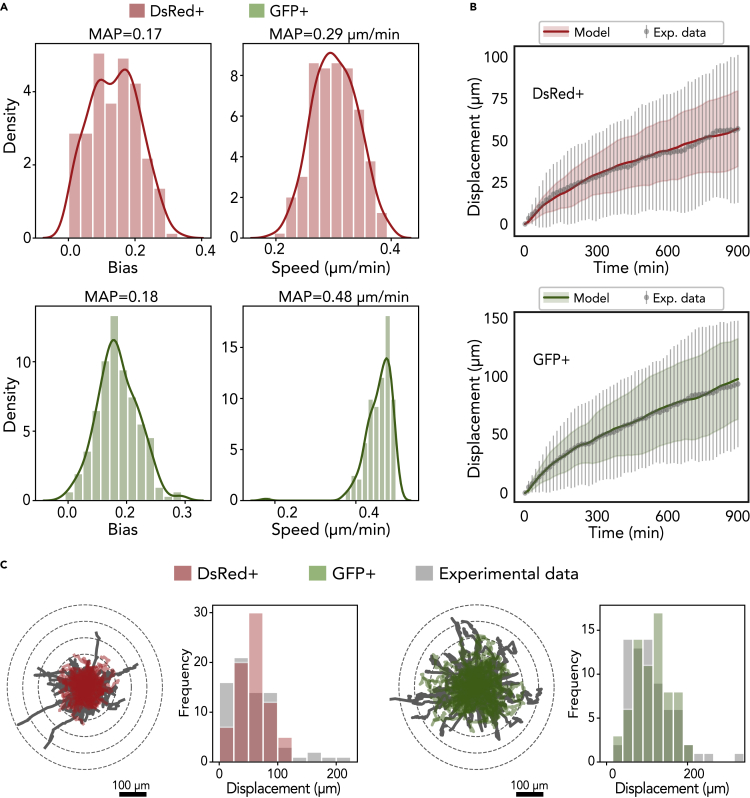
Calibration of cell migration in the model (A) Frequency histograms of the posterior distributions for *b* (migration bias) and *s* (migration speed) for DsRed+ cells (labeled red) and GFP+ cells (labeled green) using the LF-MCMC method. (B) Longitudinal changes in displacement (mean and standard deviation) obtained by simulating the model with the MAP (*ℓ*_2_ norm of the residuals between the data average and the simulated values are approximately 12.9 *μm*). (C) Trajectories of all cells (74 cells DsRed+ and 69 cells GFP+) over 15 hr and frequency histogram of the displacement, in which the gray graphs are the biological data and the red and green graphs are associated with the DsRed+ and GFP+ cells, respectively.

We calibrated our model based on the cell motility experiments. To extract the displacement profile of these cells, we simulated the random migration tracks of 100 cells seeded at the same initial position. We modeled each cell's migration independently to focus on the distribution of individual cell tracks. We set the motility persistence time (*τ*) to 15 min to match the experimental sampling frequency, and we calibrated the speed (*s*) and the bias (*b*) for DsRed+ and GFP+ cells. By applying Bayesian inference with ABC methodology, in particular, the likelihood-free Markov chain Monte Carlo (LF-MCMC) method (Marjoram et al., 2003), we obtained the posterior distribution of (*b, s*) conditioned to the experimental data *y*_*o*_ for the simulated displacement of the DsRed+ and GFP+ cells (Figure 3A).

According to the distributions obtained, we extracted the maximum *a posteriori* probability (MAP) of speed and bias, and we recomputed the simulated cell tracks using these estimated parameters. Finally, we compared the simulated displacements and trajectories with the experimental data and verified that our model was effective at recapitulating the biological observations (Figures 3B and 3C). Moreover, the average displacement obtained by the model closely approximated the observational data for both cells types; specifically, the residual for DsRed+ and GFP+ cells was 12*.*92 *μm* and 12*.*86 *μm*, respectively. The cell trajectories have a similar profile when compared with the experimental data; however, trajectories with greater displacement were observed in the experimental data, which might suggest that a subpopulation of GFP+ cells have a stronger response to migration stimuli.

### Computational model captures diverse spatial-temporal dynamic patterns

To study the effects of the enhanced migratory phenotype of the GFP+ cells on the spatiotemporal progression of the tumor without the confounding complexities of *in vivo* models, we designed a computational model for virtual thought experiments (Macklin, 2017, 2019). In our virtual system, we simulated a 3-mm square (2D) or cube (3D) with an initial tumor comprised entirely of DsRed+ cells. We arranged the cells at the center of the domain in a packed disc with a radius of 0.25 mm. We then applied the parameters calibrated in the previous section, along with other parameters defined according to literature, or estimated as specified in Table S1. We used this virtual system to assess the impact of varying the simulation rules that correspond to hypotheses on cell phenotypic responses to hypoxic conditions. Because there is uncertainty in how long a GFP+ cell can maintain its migratory phenotype after leaving a region of hypoxia, we defined a parameter for phenotypic persistence time (*T*_*p*_) and analyzed the impact of its variation on the dynamics of tumor evolution. We first assessed the impact of phenotypic persistence when hypoxic (GFP+) cells had the calibrated migrational bias (*b* = 0.1718) under three sets of hypotheses:

1. **No phenotypic persistence (***T*_*p*_ = 0**):** This represents the hypothesis that GFP+ cells do not show phenotypic persistence; they immediately resume a normoxic, nonmigratory phenotype upon leaving hypoxic tissue regions.
2. **Transient phenotypic persistence (**0 *< T*_*p*_*<* ∞)**:** This represents the case where a hypoxic cell maintains its hypoxic migratory phenotype for certain duration (*T*_*p*_) after leaving hypoxic regions. We fixed *T*_*p*_ = 50 *hr.*
3. **Permanent phenotypic persistence (***T*_*p*_ = ∞**):** This represents the scenario where hypoxic cells maintain their migratory phenotype permanently.

In this first virtual experiment, we found that increasing *T*_*p*_ promoted the mixing of DsRed+ and GFP+ cells along the hypoxic boundary region and in the viable rim. Furthermore, no GFP+ cells were able to escape through the viable rim to invade the surrounding tissue (Figure S2).

Because this does not match our *in vivo* observations where GFP+ cells frequently invade the surrounding tissues and seed distant micrometastases, we surmise that additional behavioral rules are needed for the GFP+ cells. Phenotypic persistence in the absence of strong directional cues for migration cannot drive invasion in the computational model system.

We next varied the motility bias (*b*) of the GFP+ cells and set *T*_*p*_ to 0 to assess whether changes in the randomness of migration alone could drive invasion of GFP+ cells. We evaluated the changes associated with either partially Brownian (0 *< b <* 1) or completely polarized (*b* = 1) movement along the oxygen gradient ∇σ. We found that increasing *b* caused the overall tumor to spread more quickly, allowing hypoxic cells to escape prior to necrotic death. However, none of these cells were able to completely escape the viable tumor region to begin invasion. Thus, increased directionality of cell migration alone cannot explain tissue invasion (Figure S3).

**Figure 4:**
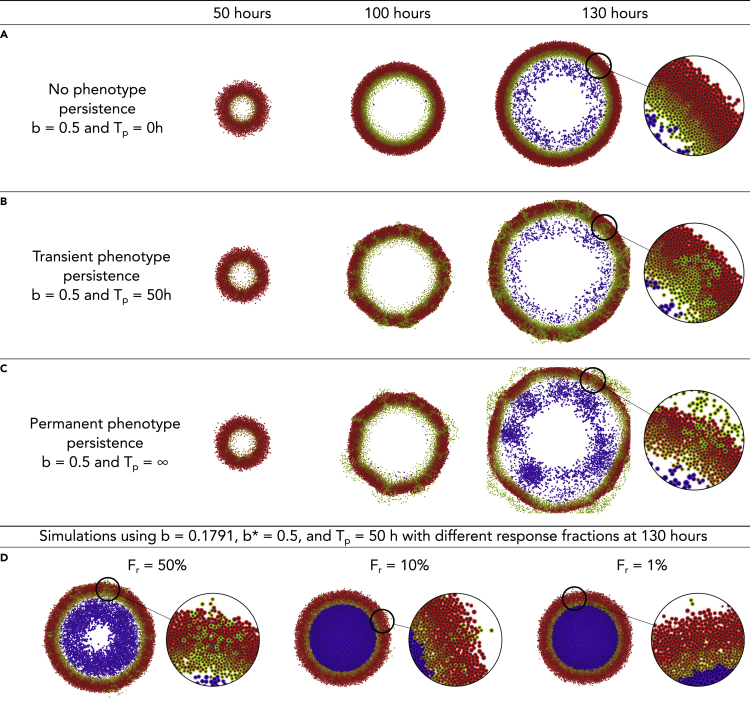
Impact of phenotypic persistence for directed migration (A) Increasing migrational bias (*b*^∗^ = 0.5) without phenotypic persistence (*T*_*p*_ = 0) does not drive invasion of GFP+ cells outside the tumor. (B) Under intermediate phenotypic persistence (*T*_*p*_ = 50 *hr*), GFP+ invasive “plumes” form inside the normoxic DsRed+ zones. Some GFPp + cells escape to invade the surrounding tissue. (C) Escape of GFP+ cells increases under permanent phenotypic persistence (*T*_*p*_ = ∞). (D) For transient phenotypic persistence (*T*_*p*_ = 50 *hr*) and intermediate migrational bias (*b*^∗^ = 0.5), decreasing the number of “responder” cells to 50%, 10%, and 1% gradually inhibits the formation of invasive plumes, although GFP+ cells continue to escape the tumor.

We then investigated whether the combination of increased migrational polarization (increased migration bias *b*) and phenotypic persistence (*T*_*p*_ *>* 0) could lead to tissue invasion by GFP+ cells. As noted, increasing migrational bias (*b* = 0*.*5) with no phenotypic persistence (*T*_*p*_ = 0) did not lead to GFP+ invasion beyond the tumor edge. Instead, the tumor grew radially, and it was composed of three homogeneous layers of necrotic, GFP+, and DsRed+ cells (Figure 4A). However, when we introduced transient (*T*_*p*_ = 50 *hr*) or permanent (*T*_*p*_ = ∞) phenotypic persistence, we found that GFP+ cells formed “plume” structures that invaded into the well-oxygenated DsRed+ regions of the tumor (Figures 4B and 4C). For sufficiently long phenotypic persistence times, we found that GFP+ cells eventually migrated through the entire DsRed+ tumor region to invade the surrounding tissue (see *t* = 130 *hr* in Figure 4B [*T*_*p*_ = 50 *hr]* and Figure 4C [*T*_*p*_ = ∞]). Thus, a persistence hypoxic migratory phenotype – when coupled with sufficiently directed migration – is sufficient to drive GFP+ cell invasion through the nonhypoxic DsRed+ tumor regions and into the surrounding tissue.

Because our experimental observations showed significantly higher displacement for a small fraction of GFP+ cells in the trajectory analysis, we hypothesized that invasion may be driven by a smaller subpopulation of “responder” cells: GFP+ cells that are capable of responding to hypoxia with a strong migratory phenotype. To computationally assess the potential impact of a responder subpopulation, we assumed that only a fraction of the GFP+ cells (*F*_*r*_) had a more directed motility along the O_2_ gradient with an elevated directional bias *b*^∗^ = 0*.*5, while all other GFP+ cells retained the lower bias of *b* = 0*.*1791 obtained from our earlier calibration work. For simplicity, all GFP+ cells shared the same value of phenotypic persistence time (*T*_*p*_ = 50 *hr*).

We found that as *F*_*r*_ decreased from 100% to 50%, 10%, and 1%, the formation of invasive GFP+ plumes was suppressed, while the extent of necrosis increased. Without directed migration, the nonresponder GFP+ cells failed to escape low-oxygen regions, leading to increased necrotic death (Figure 4D). We note that even when only 1% of GFP+ cells could respond with increased migrational bias, GFP+ cells escaped the tumor to invade the surrounding tissue.

To better automate our analysis of the qualitative behavioral responses across the (*b*, *T*_*p*_, *F*_*r*_) parameter space, we developed an image processing pipeline to extract three morphometric features (Figure S4). For each simulation, we assessed (1) whether GFP+ “plumes” were present, (2) whether GFP+ cells escaped the main tumor bulk, and (3) whether a necrotic core was present. In our analysis, we found that these Boolean features were robust to stochastic variations in the simulations (e.g., from choosing a different random seed value). See the supplementary materials for an example where we show these features for several stochastic replica on a parameter set (Figure S5).

This automated analysis further confirmed that increasing phenotypic persistence time (*T*_*p*_) and motility bias (*b*^∗^) are associated with the formation of invasive plume structures and metastatic cell escape from the tumor bulk. Moreover, in scenarios with a substantial bias (*b*^∗^ *>* 0*.*5), decreasing the fraction of responders (*F*_*r*_) is associated with increased necrosis and smaller tumor radii (Figures S6–S8). On the other hand, in scenarios of null bias (*b*^∗^ *<* 0*.*1), a cell would have a low probability of leaving the hypoxic region, preventing the formation of post-hypoxic plumes (Figures S7 and S8).

### Computationally predicted hypoxic patterns are observed *in vivo*

We performed a parameter space exploration using the simulation model system to assess the emergent tumor behaviors under a variety of hypotheses on migration, phenotypic persistence, and heterogeneity in hypoxic response. For several conditions with phenotypic persistence (*T*_*p*_ *>* 0), increased migrational bias (increased *b*^∗^ for GFP+ cells), and heterogeneous hypoxic responses (*F*_*r*_ *<* 100%), the model predicted the emergence of GFP+ plumes into DsRed+ well-oxygenated regions and the formation of substantial necrotic cores, for instance, see in Figure 4, adopting *T*_*p*_ = 50 *hr*, *b*^∗^ = 0*.*5, and *F*_*r*_ = 50%. These computationally predicted features are frequently observed in our *in vivo* tissue sections, where a necrotic core and GFP+ plumes were visible.

**Figure 5:**
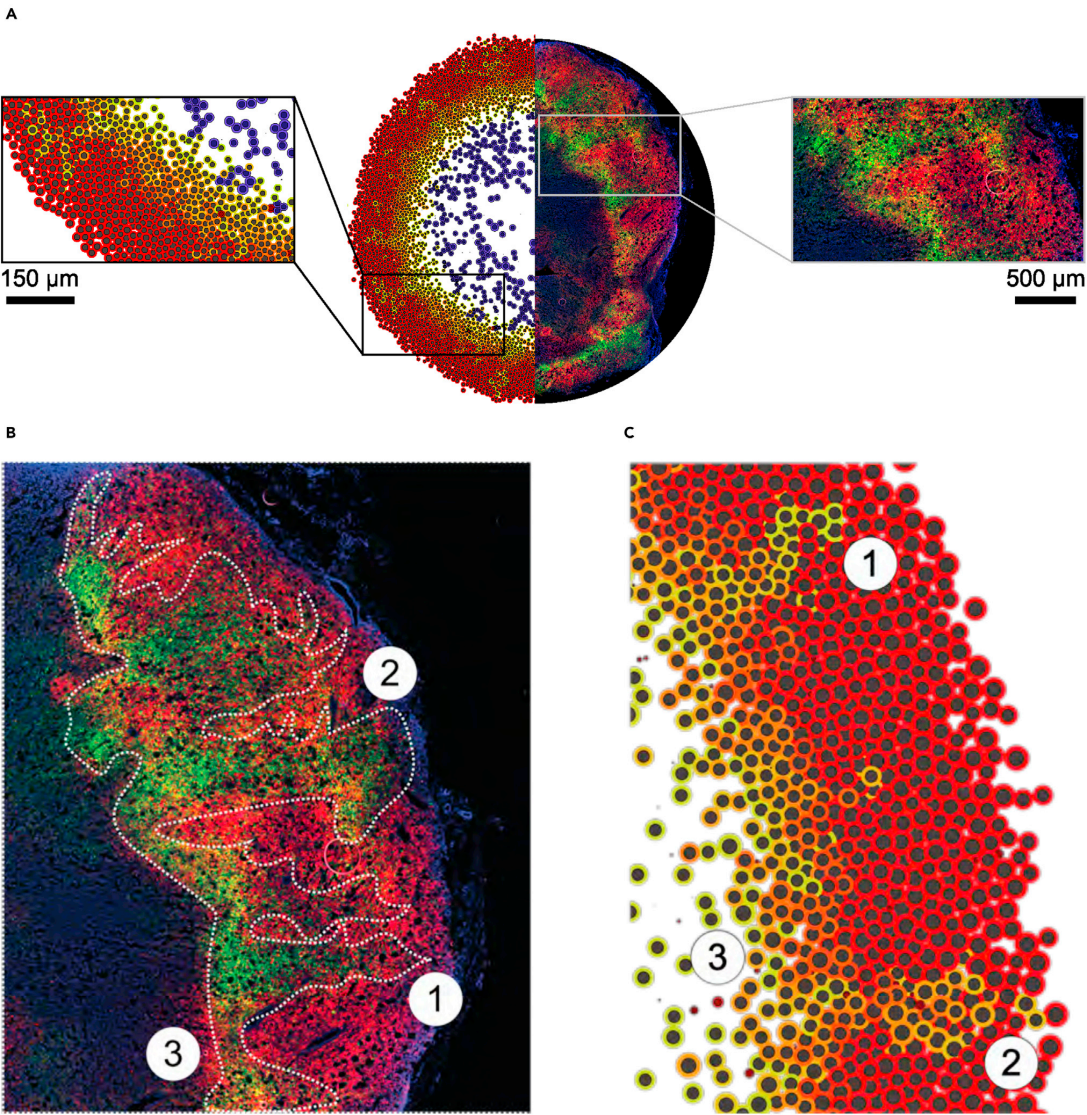
Computational model predicts the formation of post-hypoxic plumes (A) Comparison of computational output to a 2D tumor section. (B) Zoom of an orthotopic tumor section. Dashed white outline highlights post-hypoxic plumes. (C) Zoom of a computational output using *T*_*p*_ = 50 *hr*, *b*^∗^ = 0.5, and *F*_*r*_ = 50%. Numbers show analogous structures in both scenarios.

To further determine how well the computational model recapitulated the tumor biology, we visually compared a 2D tumor tissue section with our model output (Figure 5A). Strikingly, our model accurately predicted GFP+ invasive structures (Figures 5B and 5C). More specifically, we observed well-defined persistent GFP+ plumes (1), as well as a second type of plume (2) that is larger and contains a mix of GFP+ and DsRed+ cells. Furthermore, the computational model also predicted DsRed+/GFP+ regions adjacent to the necrotic core (3) that were also observed experimentally. In both the simulation and experimental models, these double-positive cells begin to express GFP but the DsRed protein is not fully degraded, indicating that these cells are currently hypoxic. All together, the data suggest that our computational model adopting *T*_*p*_ = 50 *hr*, *b*^∗^ = 0*.*5, and *F*_*r*_ = 50% most faithfully recapitulates the spatial localization of GFP+ cells *in vivo* during tumorgenesis, highlighting the formation of persistent invasive plumes.

### Invasive plumes are exclusively driven by post-hypoxic cells

**Figure 6:**
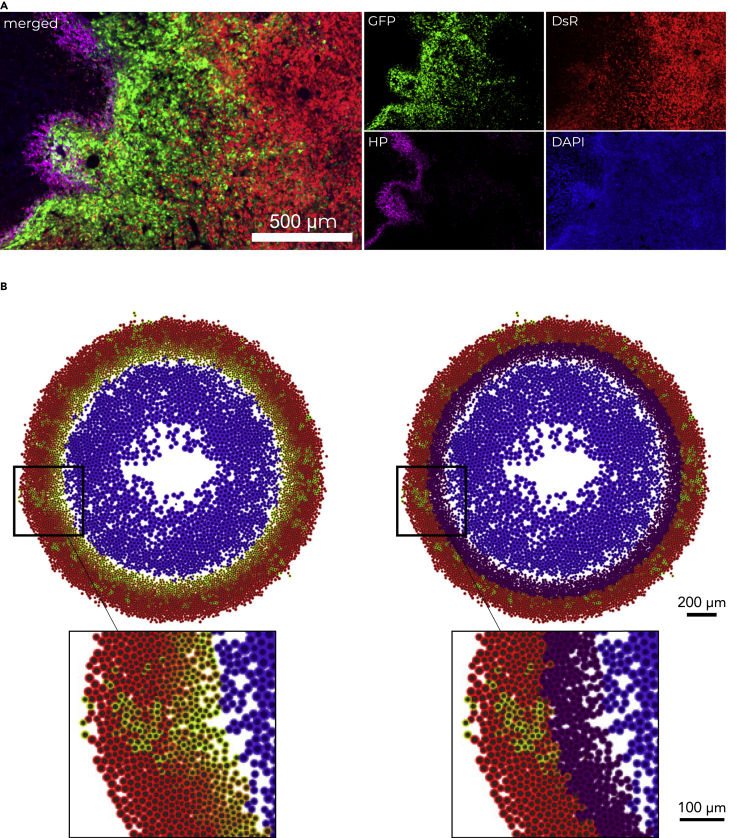
Comparison of Hypoxyprobe staining with hypoxia fate mapping system (A) Fluorescent image of orthotopic tumor section stained with Hypoxyprobe (HP). (B) Computational output without (left) and with (right) Hypoxyprobe (purple) marker.

The GFP+ plumes appeared to extend into tumor regions with higher O_2_ content. Thus, we hypothesized that the GFP+ cells localized within the invasive plumes were no longer experiencing hypoxia, but rather were cells that had been previously hypoxic and were now reoxygenated. To experimentally test this, we utilized Hypoxyprobe, a well-established antibody for the detection of hypoxic regions in tissue. Dual imaging of Hypoxyprobe and GFP expression allowed us to differentiate hypoxic cells (Hypoxyprobe+) from post-hypoxic cells (GFP+/Hypoxyprobe-) *in vivo*. While post-hypoxic cells (GFP+/Hypoxyprobe-) migrated into DsRed+ regions generating plumes, hypoxic cells (Hypoxyprobe+) localized adjacent to the necrotic core (Figure 6A). To visualize the hypoxic and post-hypoxic phenotypes in the computational model, we labeled the simulated cells localized alongside the perinecrotic region as Hypoxyprobe+ (Figure 6B). Remarkably, the computational model accurately recapitulated the observed biological differences. Together, these data suggest that the experimentally observed GFP+ invasive structures are composed exclusively of post-hypoxic cells (GFP+/Hypoxyprobe-), and thus, we termed them invasive post-hypoxic plumes.

### Computational model recapitulates intratumoral hypoxic patterns in 3D

**Figure 7:**
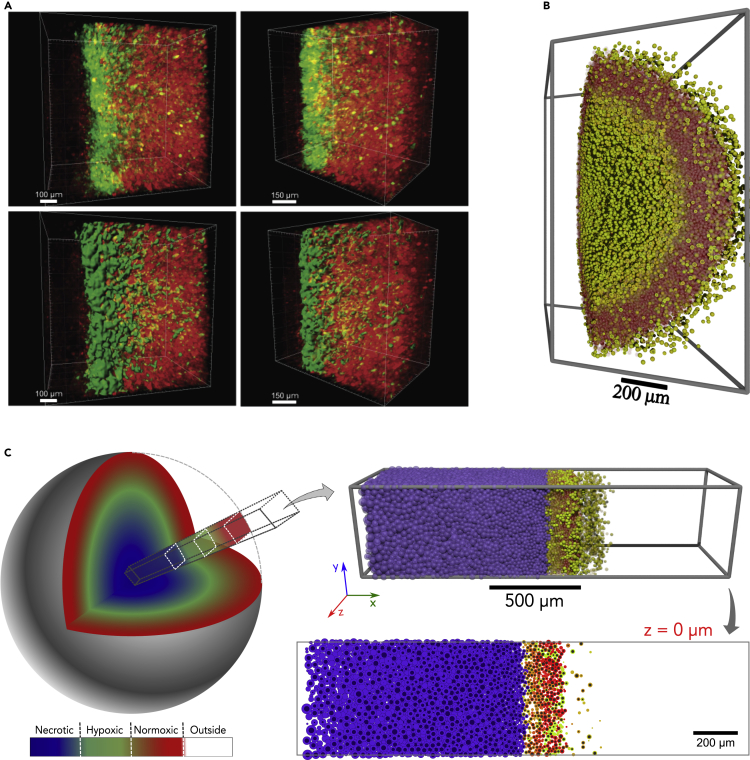
Computational model recapitulates intratumoral hypoxic patterns in 3D (A) Reconstruction of cleared tumor section in the perinecrotic region. Raw reconstruction (top) and surface rendering of GFP+ signal (bottom). (B) Small spheroid generated by the 3D computational model. (C) A 3D simulation of a radial core section in a large tumor with low surface curvature, showing the same intratumoral hypoxic pattern. The 3D core simulation is consistent with earlier 2D predictions, shown here for comparison.

We further tested whether the computational model's predictions would extend to 3D by comparing simulations to a 3D volume of tumor tissue. A 3D tumor (approximately 1*,*100 × 800 × 500 *μm*^3^) was reconstructed after CUBIC-tissue clearing, where the native fluorescence was imaged by confocal microscopy in a z stack. The surface rendering of the 3D-reconstructed image demonstrated the presence of post-hypoxic plumes (Figure 7A). However, initial 3D simulations (using the same parameter values as in 2D: *T*_*p*_ = 50 *hr*, *b*^∗^ = 0*.*5, and *F*_*r*_ = 50%) predicted less well-defined post-hypoxic GFP+ plumes than we observed experimentally (Figure 7B). We hypothesized that this apparent discrepancy arose due to differences between the computational and experimental geometries: the 3D computational spheroid had a relatively small radius and hence high surface curvature, which contributed to dispersal of migrating GFP+ tumor cells. Under this hypothesis, 3D simulations would better match experimental observations and earlier 2D computational predictions for larger tumors in regions with flatter surface curvature.

We tested this by simulating a radial section of a larger 3D tumor with flatter surface curvature: we modified the simulation domain to a 0*.*5-mm^3^ “core” (2 *mm ×* 0*.*5 *mm ×* 0*.*5 *mm*) with no cell or substrate flux in the nonradial (*y* and *z*) directions and with free cell and substrate flow in the radial (*x*) direction (see this geometry in Figure 7C). As hypothesized, necrotic regions and post-hypoxic GFP+ plumes were better defined in this 3D geometry, and 2D cross sections were consistent with earlier 2D simulation results (Figure 7C).

Taken together, the computational model accurately predicted the localization of cells exposed to hypoxia within a tumor and anticipated the formation of post-hypoxic plumes both in 2D and 3D. According to our model using *T*_*p*_ = 50 *hr*, *b*^∗^ = 0*.*5, and *F*_*r*_ = 50%, the resulting phenotype depends upon a balance of phenotypic persistence time, the fraction of responders, and migrational bias of the cellular movement, supporting the hypothesis of an enhanced migratory hypoxic/post-hypoxic phenotype.

## Discussion

We combined an approach to fate map cells that experience intratumoral hypoxia with advanced imaging and mathematical modeling to predict the dynamics of hypoxic and post-hypoxic cells during tumor progression. Investigating GFP+ (hypoxic/post-hypoxic) and DsRed+ (normoxic) cells in tumor tissue sections revealed that both cell populations have similar proliferation rates. On the other hand, GFP+ cells are more migratory *ex vivo* than DsRed+ cells. Using these characteristics and the O_2_ distribution in a tumor, we modeled the dynamics of GFP+ cells with three key parameters: phenotypic persistence time (*T*_*p*_), fraction of responders (*F*_*r*_), and directional bias of the migrating GFP+ responders (*b*^∗^).

Our computational studies best recapitulated *in vivo* biological observations of (1) localization of GFP+ and GFP/dsRed double-positive cells to the perinecrotic regions of the tumor, (2) migration toward more oxygenated regions, (3) invasive GFP+ post-hypoxic plumes, (4) substantial necrosis, and (5) escape of GFP+ cells from the tumor bulk, when we defined phenotypic persistence (*T*_*p*_ = 50 *hr*), increased directional bias of migrating GFP+ cells (*b*^∗^ = 0*.*5), and included heterogeneity in response to hypoxic stimuli (*F*_*r*_ = 50%). Furthermore, by examining the Hypoxyprobe marker for currently hypoxic cells both *in vivo* and *in silico* models, we determined that the GFP+ invasive structures were exclusively driven by post-hypoxic cells. The results support the conclusion that post-hypoxic plumes form due to a persistent migratory phenotype that develops when a cell becomes hypoxic and persists even when the cell is reoxygenated.

The *ex vivo* spheroid model used to determine cell motility consisted of measuring the displacement of tumor-derived cancer cells (DsRed+ and GFP+) at the invasive front of spheroids embedded in a 3D collagen matrix. We observed enhanced speed, total displacement, and persistent time of GFP+ cells, suggesting that both migration and invasion contribute to cell motility in this model. In these experiments, we also observed that a fraction of GFP+ cells showed significantly higher displacement than the remaining GFP+ cells, suggesting that only a subset of GFP+ cells acquire an increase in migration. To account for this in the model, we varied the directional bias for cell migration (*b*^∗^) for this responder subpopulation and observed that higher values (*b*^∗^ 0*.*5) promoted the formation of post-hypoxic plumes. This phenomenon was surprisingly robust to perturbation: post-hypoxic plumes were consistently observed even when only 50% of the cells were responders (*F*_*r*_ = 50%). However, experimentally observed necrotic materials could only accumulate in the computational model when not all GFP+ cells were responders (*F*_*r*_ *<* 100%). There are several potential biological explanations for this finding. First, the spatial localization of a cell within the O_2_ gradient in the tumor may cause heterogeneity in the level of migratory response. For example, cells localized closer to the necrotic core in a tumor experience lower O_2_ concentrations (<1%) and have higher levels of HIF expression and therefore higher levels of HIF-dependent pathways that promote cell migration and invasion, such as RhoA-ROCK (Gilkes et al., 2014), ITGA5 (Ju et al., 2017), ADAM12-HB-EGF-EGFR (Wang et al., 2021b), Notch (Chen et al., 2010), and miR-219-SMC4 (Chen et al., 2019), among others. Additionally, the observed invasion pattern suggests that cells localized in the invasive front must overcome extracellular matrix resistance by opening tracks, which would facilitate the invasion/migration of cells that follow, increasing their bias in a leader-follower-like manner (Haeger et al., 2015; Khalil and Friedl, 2010; Mayor and Etienne-Manneville, 2016).

We explored a range of the phenotypic persistence time(s) and found that any sufficiently high value (50 hr or more in our studies) would result in the formation of GFP+ plumes. This suggests that the formation of post-hypoxic plumes is associated with a persistent phenotype that is maintained in GFP+ tumor cells for a considerably long duration. Although HIF-1 protein levels return to baseline within minutes after reoxygenation to room air (≈20% O_2_) (Jaakkola et al., 2001; Semenza and Wang, 1992), the time required for the protein products of HIF-regulated genes to return to baseline will depend upon the half-life of the protein. Moreover, the hypoxic cells within a tumor encounter a slower reoxygenation as they migrate toward the oxygenated areas and experience a lower O_2_ gradient (1%–6% O_2_) causing prolonged HIF signaling when compared with *in vitro* studies. Therefore, post-hypoxic cells within *T*_*p*_ = 50 *hr* will retain an increased level of HIF-target gene and protein expression which could, in part, explain the lengthy persistence time. Indeed, we previously demonstrated that cells exposed to intratumoral hypoxia, but not short-term *in vitro* hypoxia, retain the increased expression of a subset of hypoxia-regulated genes, even upon reoxygenation and long-term culture (Godet et al., 2019). Further studies would be necessary to establish whether the expression of one or multiple genes plays a causal role in phenotypic persistence. However, it is tempting to speculate that the “hypoxia memory” genes LOX or DNAH11 could be implicated, as they can regulate cell movement (Lai et al., 2016; Payne et al., 2005).

In the future, we plan to extend the mathematical model to include cell signaling and to more accurately match the dynamics such as O_2_ distribution, proliferation, and tissue and cell mechanics. The implementation of new calibration protocols to leverage high-performance computing (HPC) and ABC techniques will be crucial to simultaneously evaluate all these parameters. Ultimately, we will also aim to advance our single-cell tracking and plan to include additional cell cycle markers. The labeling and characterization of blood vessels in tumor sections (CD31 or dextran) could be used to assess angiogenesis. In addition, the contribution of the extracellular matrix and the leader-follower dynamics will be important to consider. The HIF response could be incorporated in our computational model by building a network of protein interactions and considering their half-life times, which could further strengthen our understanding of hypoxic/post-hypoxic phenotypes.

In summary, we developed a computational model based on the characterization of normoxic, hypoxic, and post-hypoxic cancer cell phenotypes that accurately predicted the formation of structures that we termed “invasive post-hypoxic plumes.” Although it is well established that hypoxia promotes migration and invasion *in vitro* during exposure to hypoxia, we show that post-hypoxic cells retain a migratory phenotype that contributes to plume formation. While at the macroscopic level the invasive plumes *appear* to be driven by collective invasion (a coordinated and guided migration) (Yang et al., 2019), our computational findings demonstrate that these structures can spontaneously arise without any direct cell-cell coordination. The results show the potential for combining experimental approaches with computational modeling: the hypoxia fate mapping system rendered the previously unobserved post-hypoxic plumes visible, computational modeling allowed us to probe the underlying dynamics to suggest hypotheses that can generate the structures, and targeted experimental analyses were able to confirm the computational predictions.

### Limitations of the study

Interestingly, we noticed that the observed computational plumes seem narrower than the biological ones. While our *in vivo* tumors reach diameters of 10 mm, our computational model reached a radius of about 0.6 mm. Therefore, it could be expected that larger tumors would give origin to larger plumes. The computational time to model a 3D tumor with a diameter of 10 mm is not feasible. Moreover, imposing dynamics of leader-follower/collective invasion that can potentially be happening *in vivo* could drive larger computational plumes. Lastly, the *in vivo* timescale is larger than the computational one, and therefore, proliferation of GFP+ cells might be contributing to larger plumes as well. Overall, our computational model recapitulates GFP+ plume formation, and perhaps larger tumors and longer time scales could contribute to generate larger plumes.

It is important to note that the MDA-MB-231 orthotopic tumors modeled in this work are uniformly radial and homogeneous. To test if our observation of GFP+ plume formation would still occur in heterogeneous environments, we performed a simulation varying the location and number of O_2_ sources. In order to avoid nonphysical behavior, we imposed restrictions on cell proliferation and migration in regions of higher cell density. In this heterogeneous scenario, GFP+ plumes were still observed, mainly around more oxygenated regions (Figure S9). This suggests that our observation of GFP+ plume formation is robust and O_2_ dependent and not simply a consequence of radial growth. Human breast tumors have a slower growth rate and therefore have additional time for angiogenesis to occur. Angiogenesis results in a random distribution of functional vasculature within the tumor that can cause heterogeneous O_2_ gradients (Vaupel et al., 2007). Together, these factors promote multiple microregional O_2_ gradients that exist within a larger O_2_ radial gradient across the tumor. It would be interesting to incorporate an intermittent subscale O_2_ distribution, by modeling angiogenesis and blood supply dynamics. Transient changes in O_2_ gradients that occur locally have been reported but occur in the range of 1.3–2.6% (Matsumoto et al., 2010). However, the overall O_2_ distribution within a viable rim of 2 cm in a large tumor ranges from 0 to 6% O_2_ (Godet et al., 2019), suggesting that the radial O_2_ gradient observed in our experimental tumors is the largest contributor to plume formation. Therefore, we hypothesize that the model would reveal similar radial O_2_ gradients with GFP+ plumes formation on both macroscales and microscales.

## STAR★Methods

### Key resources table

**Table:** 

REAGENT or RESOURCE	SOURCE	IDENTIFIER
**Antibodies**		

Alexa Fluor 647 mouse anti-ki-67	BD Biosciences	Cat #558615; RRID:AB_647130
Alexa Fluor 647 mouse	Invitrogen	Cat #A21237; RRID:AB_2535806

**Chemicals, peptides, and recombinant proteins**		

Formalin	Sigma-Aldrich	HT501128
OCT	Fisher Scientific	23-730-571
Sucrose	Sigma-Aldrich	57-50-1
DAPI	Sigma-Aldrich	D9542
BSA	Sigma-Aldrich	A9647
Collagen rat type I	Corning	354236
Urea	Sigma-Aldrich	U5378
Triethanolamine (Quadrol)	Sigma-Aldrich	122262
Triton X-	Sigma-Aldrich	X100

**Critical commercial assays**		

Hypoxyprobe™ Kit	Hypoxyprobe, Inc	HP1-XXX

**Experimental models: Cell lines**		

MDA-MB-231	ATCC	HTB-26™

**Experimental models: Organisms/strains**		

Mice: NOD-SCID Gamma (NSG)	The Jackson Laboratory	005557

**Recombinant DNA**		

4xHRE-MinTK-CRE-ODD	Addgene	141147
CMV-loxp-DsRed-loxp-eGFP	Addgene	141148

**Software and algorithms**		

PhysiCell	Ghaffarizadeh et al., 2018	http://physicell.org/
xml2jupyter	Heiland et al., 2019	https://github.com/rheiland/xml2jupyter
nanoHUB platform	Madhavan et al., 2013	https://nanohub.org/
PhysiCell-povwriter	Simple cutaway POV-Ray (2019)	https://github.com/PhysiCell-Tools/PhysiCell-povwriter
POV-Ray	Persistence of Vision Pty. Ltd. (2004)	http://www.povray.org/
OpenCV	Open Source Computer Vision Library. 2015	https://pypi.org/project/opencv-python/

**Other**		

Computational model in C++	This paper	https://github.com/PhysiCell-Models/breastcancer-hypoxia
Online interactive version of model	This paper	https://nanohub.org/tools/pc4tumorhypoxia
Image classifier	This paper	https://github.com/heberlr/Image_Classifier
Calibration of parameters and associated figures	This paper	https://github.com/heberlr/Motility_Calibration

### Resource availability

#### Lead contact

Further information and requests for resources and reagents should be directed to and will be fulfilled by the Lead Contact, Paul Macklin, PhD (macklinp@ui.edu).

#### Materials availability

The vectors used to generate the hypoxia fate mapping system in this article are available at Addgene (#141147 and #141148).

### Experimental model

#### Cell culture

Mycoplasma-free human breast cancer cell line MDA-MB-231 was obtained from the American Type Culture Collection (ATCC) and maintained in DMEM (Dulbecco's Modified Eagle Medium; Sigma-Aldrich) with 10% FBS (fetal bovine serum; Corning) and 1% penicillin/streptomycin (Invitrogen). An InvivO_2_ workstation (Baker) with an ICONIC (Baker) electronically controlled gas-mixing system was used to achieve hypoxic conditions in a controlled environment at 37°C and 75% humidity and balanced at 0.5% O_2_, 5% CO_2_, and 94.5% N_2_.

#### Animal model

Female 5- to 7-week-old NOD-SCID Gamma (NSG) mice were used according to protocols approved by the Johns Hopkins University Animal Care and Use Committee. Mice were anesthetized by the intraperitoneal (i.p.) injection of 100 mg/kg Ketamine, 16 mg/kg Xylazine, Vet One. MDA-MB-231 hypoxia fate mapping cells (2 × 10^6)^ were injected into the mammary fat pad (MFP) closest to the second nipple. Mice were i.p. injected with 1.25 mg of pimonidazole in saline (12.5 mg/mL) (Hypoxyprobe-1) 1 hr prior to sacrificing. Tumors were excised at various time points, formalin fixed (Sigma-Aldrich) for 1 hr, saturated in 30% sucrose (Sigma-Aldrich) at 4°C overnight, embedded in OCT media (Fisher Scientific), frozen in liquid nitrogen, sectioned via a cryotome CM1100 (Leica), and mounted onto Superfrost Plus microscope slides (Fisher Scientific). Tumor tissue sections were stained with DAPI (4′,6-diamidino-2-phenylindole) (1:1000 for 15 min, RT) and mounted with anti-fade solution. To assess the entire cross section of the tumor, slides were imaged with an Olympus (UPLFLN 4×) objective using Cytation 5 microscope (BioTek Instruments). Multiple image tiles were linearly stitched with Gen5 Software (BioTek Instruments).

### Method details

#### Hypoxia fate mapping system

The loxp-DsRed-loxp-eGFP sequence was amplified by polymerase chain reaction (PCR) from the plasmid pMSCV-loxp-DsRed-loxp- eGFP-Puro-WPRE (#32702, Addgene). This sequence was then ligated into the pENTRA1 vector, and the Gateway System TM (Invitrogen) was used to recombine the pENTR1A shuttle vector with pLenti CMV/TO Zeo DEST (644-1) (#17294, Addgene) generating lentiviral vector 1. The sequence GTGTACGTG (1HRE) spaced with random 5 base pairs of nucleotides was used to generate tandem copies of 4 HREs that were directly synthesized (IDT) as gBlocks (Coralville). The CRE-ODD nucleotide sequence was also independently synthesized as a gBlock (IDT). The pRRLSIN.cPPT.PGK-GFP.WPRE (#12252, Addgene) was digested to remove the PGK promoter and EGFP cassette. In-Fusion Cloning (Clonetech) was used to ligate the 4-HRE gBlock, the *β* -globin gBlock, and the CRE-ODD gBlock into the linearized pRRLSIN.cPPT.PGK-GFP.WPRE vector to generate vector 2. Finalized lentiviral vector 1 encoding CMV-loxp-DsRed-loxp-eGFP (Addgene #141148) or lentiviral vector 2 encoding 4xHRE-MinTK-CRE-ODD (Addgene #141147) was cotransfected with plasmid psPAX2 (#12260, Addgene) and plasmid pMD2.G (#12259, Addgene) into 293T cells using Polyjet (SL10088, Signagen). Filtered viral supernatant generated from lentiviral vector 1 was collected 48 hr post transfection and added to MDA-MB-231 cells with 8 *μ*g/mL polybrene (Sigma–Aldrich) overnight. After 24 hr in fresh media, zeocin (Invitrogen) was added to the medium of cells for selection (100 *μ*g/mL). Following selection, cells were transduced with lentivirus from lentiviral vector 2 encoding 4xHRE-MinTK-CRE-ODD. The cell lines were sorted, single-cell cloned, and screened by image analysis and flow cytometry. More details can be found in the study by Godet et al. (2019).

#### Oxygen measurements

*In vivo* O_2_ measurements were conducted on mouse tumors using a fixed-needle (0.9 mm/230 *μ*m) REDFLASH mini-sensor (FireStingO_2_, Pyroscience) mounted on a manual micrometer. The probe was inserted approximately 2 mm into the tumor, and after recording the hypoxic level, it was slowly retracted and O_2_ measurements were recorded at 0.5 mm intervals until reaching the tumor edge. The needle probe was carefully washed and calibrated to atmospheric O_2_ before each measurement.

#### Immunofluorescence staining

Tumor tissue sections were washed 3x with PBS-T (PBS, 0.1% Tween) and briefly air-dried. Using a hydrophobic pen (Dako, Angilent), a circle was drawn around the tumor sections, and they were then incubated with 1% Triton X- at room temperature for 10 min in the dark. After a new wash with PBS-T, the tissue sections were incubated with 2% bovine serum albumin (BSA) at room temperature for 30 min in the dark. The Alexa Fluor 647 Mouse anti-Ki-67 antibody (BD Biosciences, #558615) was used at 1:50 dilution in BSA at room temperature for 1.5 hr in the dark. For Hypoxyprobe-1 staining, slides were incubated overnight at 4°C with the primary antibody (dilution at 1:50). On the following day, mouse (#A21237) secondary Alexa Fluor 647(Invitrogen) antibody was used at 1:1000 dilution for 1.5 hr at RT. New PBS-T wash was followed by staining with DAPI at 1:1000 dilution in PBS at room temperature for 15 min in the dark. Slides were washed and mounted with anti-fade solution. Images were taken with an Olympus (UPLFLN 10×) objective using Cytation 5 microscope (BioTek Instruments). Image analysis was performed by determining the ratio of Ki67+ nuclei over total nuclei in each RFP and GFP channels using the image calculator tool available on ImageJ.

#### Spheroid model

Cell spheroids were formed in round-bottomed 96-well tissue culture plates. Briefly, a mixture of 1 × 10^4^ cells, 80–90% unlabeled cells and 10–20% hypoxia fate-mapping cells derived from orthotopic tumors, were plated per well in spheroid formation media 1:1 DMEM (Sigma-Aldrich) and Methocult H4100 (STEMCELL Technologies). Plates were centrifuged at 1200 rpm for 7 min, twice. After 72 hr of incubation, each spheroid was transferred to a Petri dish, where they were individually isolated with the collagen solution (2 *mg/mL*) and quickly transferred to the center of a semi-cross-linked collagen gel in a 96-well plate at 37°C. After complete cross-linking, warm media was added. Following 4 days in culture, spheroids were imaged in an environmentally controlled microscope every 15 min for 16 hr using an Olympus (UPLFLN 4×) objective in Cytation 5 (BioTek Instruments). Cell trajectories were tracked using MetaMorph software to obtain x,y coordinates at each time. More details are available in the studies by Ju et al. (2017) and Valencia et al. (2015).

#### CUBIC tissue clearing

Tumor tissue clearing was achieved by following the CUBIC protocol (Susaki et al., 2015), and CUBIC-I (water 35% wt, urea 25% wt, Quadrol 25% wt, Triton X-15% wt) and CUBIC-II (sucrose 50%, urea 25%, water 15%, triethanolamine 10%) solutions were prepared prior to use. Tumors resected at day 20 were fixed in formalin at 4°C overnight. Tumors were then incubated with 1:1 CUBIC-I/water overnight at 37°C and 60 RPM in an orbital shaker. On the following day, tumors were transferred to CUBIC-I and kept at 37°C and 60 RPM in an orbital shaker until desired clearness was achieved (approximately 2.5 weeks), with CUBIC-I being replaced every other day. Once clearing was satisfactory, tumors were transferred to CUBIC-II for 2 days. Tumors were imaged in mineral oil using confocal microscopy to obtain z-projections of tumor sections with approximately 500 *μ*m depth using a 10X/0.45 PlanApo (dry, no DIC) objective in a Zeiss LSM780-FCS microscope. Z-stacks spaced at 6.3-*μ*m intervals were processed into a 3D image via Imaris version 9.2 (Bitplane), and 3D surface rendering was used to visualize the GFP+ invasive structures in 3D.

#### PhysiCell: agent-based cell modeling

The computational model was developed using PhysiCell, an open-source C++ framework allowing the construction of multicellular models at various scales in 2D and 3D (Ghaffarizadeh et al., 2018) on a broad variety of computing platforms. In this structure, each cell has its phenotypic and physical properties individually characterized through an agent-based model. Generally, cell dynamics are partially determined by diffusion of substrates such as oxygen, glucose, and growth factors in the microenvironment. To model the distribution of these substrates in the tissue environment, PhysiCell uses an open-source biological diffusion solver, BioFVM (Ghaffarizadeh et al., 2016). The coupling between these computational tools is defined by the consumption and secretion of substrates by each cell. PhysiCell has been applied to a broad variety of multicellular problems, such as oncolytic virus therapy, cancer immunology, tissue mechanics, infection dynamics and tissue damage, and drug screening, among others (Ghaffarizadeh et al., 2018; Jenner, 2019; Ozik et al., 2019). Please refer to the studies by Ghaffarizadeh et al., 2018 for further computational details and performance testing.

#### Computational model

Using our biological observations, we applied PhysiCell (version 1.6.1) to develop a mathematical model representative of tumor progression that incorporates the cell phenotype, location, and exposure to spatially dependent O_2_ concentrations. In this model, we differentiate the normoxic, hypoxic, and necrotic state of the cancer cell based on the level of pO_2_. The O_2_ concentration is distributed in the environment using the standard transport equations from BioFVM and PhysiCell (Ghaffarizadeh et al., 2016, Ghaffarizadeh et al., 2018). Here, we define the pO_2_ as σ. In regions outside the tumor, we set the O_2_ concentration to a (maximum) far-field value, denoted by $\bar{\sigma }$.

A change in phenotype from normoxic to hypoxic occurs only in response to a decrease in O_2_ availability below the threshold, σ_*H*_. When the *O*_2_ level decreases further, below a σ_*T*_ threshold, the cell is modeled necrotic (or dead). Full details on the necrosis model can be found in the study by Ghaffarizadeh et al., 2018. When a hypoxic cell migrates to a region with a high supply of oxygen, we characterized it as a post-hypoxic or reoxygenated cell. The concentration of pO_2_ also contributes to the rate of cellular proliferation in this model. We use the built-in Ki67 basic model from PhysiCell, where each cell has two states according to its current stage in the cell cycle. The first state [*Ki*67-] represents cells with absence of Ki67 expression, and the second state [*Ki*67+] represents positive expression of Ki67, in which cells have already started the proliferation process.

In our hypoxia fate mapping system, all cells begin by expressing DsRed, and upon exposure to hypoxia, they permanently switch from DsRed to GFP expression. The process of transitioning from DsRed to GFP protein expression is modeled through a system of two ordinary differential equations (ODEs), in which given a set of (normalized) genes **G** = (*G*_0_*, G*_1_) encoding the proteins, the (normalized) protein concentration in each cell is denoted by

(Equation 1) $$\frac{\mathrm{d}\left[DsRed\right]}{\mathrm{d}t}=\alpha_{0}G_{0}\left(1-\left[DsRed\right]\right)-\beta_{0}\left[DsRed\right]\left(1-G_{0}\right)$$

(Equation 2) $$\frac{\mathrm{d}\left[GFP\right]}{\mathrm{d}t}=\alpha_{1}G_{1}\left(1-\left[GFP\right]\right)-\beta_{1}\left[GFP\right]\left(1-G_{1}\right)\;$$

where *α*_*i*_ is a protein production rate and *β*_*i*_ is a protein degradation rate. Note that in this normalized model, each protein *i* decays exponentially with rate *β*_*i*_ when its gene expression is 0, and it tends toward a steady-state value of 1 when its gene expression is 1. We calibrated the production and degradation rates of GFP and DsRed, respectively, based on our experimental data. As an initial condition, we assumed that [D*sRed*](0) = 1 and [*GFP*](0) = 0. As a simplifying assumption, we define **G** = (1*,* 0) as gene expression of normoxic cells that never experienced hypoxia and **G** = (0*,* 1) as permanent gene expression of cells that have been subjected to hypoxia. It is important to note that GFP expression is permanent once cells experience hypoxia due to the gene editing mechanism (Figures 2A and 2B).

Cellular motility is characterized in the model by four intrinsic properties: persistence time (*τ*: the mean time that the cell maintains its current speed and direction); migration speed (*s*: the speed of cellular migration in the absence of other forces); the migration bias direction (**d**: the preferred direction of migration), and migration bias (*b*: a value between 0 and 1 that represents the extent that cell trajectory mimics Brownian motion). Zero bias (*b* = 0) represents purely Brownian motion, whereas *b* = 1 is completely deterministic motion along its preferred direction **d**. (Refer to the study by Ghaffarizadeh et al., 2018 for further details, or an online motility demonstrator at https://nanohub.org/tools/trmotility). In the present model, we use the O_2_ gradient **∇**σ as the preferred direction of motility for biased migration (**d** = ∇σ).

Based on our biological observations (Figures S1B and S1C), we assume that DsRed+ and GFP+ cells have identical proliferation capacity under identical conditions. In contrast, cell migration has specific characteristics for each type of cell. As seen in the biological experiments, GFP+ cells have a more pronounced migratory phenotype.

#### Approximate Bayesian computation

Model parameters were calibrated using an ABC method. ABC is a class of inference techniques based on Bayesian statistics without the explicit construction of the likelihood function, and this terminology has been used since 2002 (Beaumont et al., 2002). This approach has become quite popular and has been applied to problems of parametric inference and model selection. Particularly, this technique has been widely used in the analysis of complex problems in the biological sciences (such as systems biology, ecology, and others) (Sunnåker et al., 2013).

In a general Bayesian inference procedure, we want to estimate the parameters associated with the model, such that the observational values are matched with the model outputs. Here, we apply an ABC method proposed by Marjoram et al. (2003), named LF-MCMC. We compute an approximation of the posterior distribution of the model parameters, *π*_*LF*_ (*θ|y*_*o*_), based on an a priori distribution of the parameters, *π*(*θ*), and observational data, *y*_*o*_. Thus, an approximate version of Bayes's theorem (Sisson and Fan, 2011) is

(Equation 3) $$\pi_{LF}\left(\theta |y_{0}\right)\approx \;\frac{\pi \left(\theta \right)}{S}\sum_{s=1}^{S}\pi_{\varepsilon }\left(y_{0}|x^{s},\;\theta \right)$$

where *x*^1^*, …, x*^*S*^ are independent draws from the model *π*(*x|θ*) and *ε* is a tolerance value of the residual between the experimental data (*y*_*o*_) and the model output (*x*). It is important to note that when *ε* is small enough, using a conventional metric, the approach to posterior distribution is more accurate. However, choosing a small epsilon requires a high computational effort. In this work, for simplicity, we used the metric induced by the *ℓ*_2_ norm and estimate epsilon values based on the observational data.

#### Image classifier

We built an image Boolean classifier to define the occurrence of plumes, escaping cells, and necrotic core (see the example in Figure S4). To check whether plumes formed, we extract the green cells from the image (Figure S4A) and then select bigger contour of the cell cluster. We fit that contour by an ellipse *E*_*G*_ and by a piecewise polynomial function *F*_*G*_. Based on the distance of the vertices of the function *F*_*G*_ and ellipse *E*_*G*_, we define whether there is a plume or not, according to tolerance *ε*_*P*_ (Figure S4B). For the cell escape test, we fit an ellipse *E*_*T*_ to the tumor and search for green cells outside that ellipse, and then we verify if the sum of these cells' areas is greater than *ε*_*S*_ (Figure S4C). The existence of the necrotic nucleus in the image occurs when the area fraction of necrotic cells is greater than *ε*_*N*_ (Figure S4D). In general, the tests are defined as

$$\begin{array}{lll} Plumes: & d\left(v,E_{G}\right),v\;vertex\;of\;F_{g}\; & \begin{array}{l} True,\;if\;d\left(v,\;E_{G}\right)>\varepsilon p\\ False,\;else \end{array}\\ Cell\;escape: & A=\sum A_{i},\;A_{i}\;area\;of\;the\;GFP+\;cell\;i\;outside\;of\;E_{T} & \begin{array}{l} True,\;if\;A>\varepsilon S\\ False,\;else \end{array}\\ Necrotic\;core: & F_{N}\frac{Necrotic\;area}{Total\;area} & \begin{array}{l} True,\;if\;A>\varepsilon N\\ False,\;else \end{array} \end{array}$$

in which *d* is distance function. In this work, we used *ε*_*P*_ = 30 *px*, *ε*_*S*_ = 20 *px*^*2*^, and *ε*_*N*_ = 0*.*06. This classifier was developed in Python3 using the OpenCV and NumPy libraries (https://github.com/heberlr/Image_Classifier).

Since our model is stochastic, we conducted a study to test the capability of our model to maintain the same classification. Thus, we executed 20 replicas of our model for the following parameters: *F*_*r*_ = 50%, *b*^∗^ = 0*.*5, and *T*_*p*_ = 50*hr*. According to Figure S5, we found that 19 replicates were classified with plumes, 18 with cell escape, and all replicates without a necrotic nucleus. This experiment shows that the classification does not substantially change for the fixed parameters. After verifying this, we performed a study of the response associated with the parametric space (Figures S6–S8).

#### Simulation of heterogeneous oxygen distribution

To investigate a scenario with multiple O_2_ sources, we display the simulation of the model for the case in which we arranged oxygen sources randomly in the computational domain, adopting *F*_*r*_ = 50%, *b* = 0*.*5, and *T*_*p*_ = 50 *hr*. This simulation showed that the distribution of oxygen modified the geometry of the tumor, changing the mechanical aspect of tumor growth. In Figure S9A, we found that the constant flow of GFP+ cells toward regions of greater oxygenation (proximity to blood vessel sections) implies a rapid saturation of cell density, losing invasive “plumes" and may even generate nonphysical phenomena, such as the overlapping of cellular nuclei. Based on this observation, we added new mechanical restrictions on the dynamics of migration and proliferation extending recent modeling work (Wang et al., 2021a). We applied restrictions based on the dimensionless mechanical pressures in each cell (simple_pressure of PhysiCell). When the mechanical pressure is above a certain threshold as in regions of high cell density (10, estimated), the cell will not proliferate and will have a migration stimulus in the opposite direction. In Figure S9B, we present a simulation including negative feedback on proliferation and migration. Note that in this new formulation of the model, the invasive structures remain well defined along time evolution. Another interesting topic that could be included in this new approach would be incorporating the mechanical effects of cell intravasation into the blood vessels. Based on the mechanical pressure around the blood vessels, tumor cells would have a probability of breaking through the barrier to the basement membrane of the capillaries. This, in turn, would reduce the cell density near the vessels, allowing further proliferation and cell flux.

### Additional resources

#### nanoHUB platform

The computational model built is available for simulations on the nanoHUB platform (Madhavan et al., 2013). The nanoHUB platform is an open and free environment that offers several services aimed at the dissemination of science and engineering products. In particular, it offers a toolkit for the development of GUIs that allow greater accessibility and reproducibility of software.

## Data Availability

• The code used to simulate the model and generate the results included in the figures is avail-able at: https://github.com/PhysiCell-Models/breastcancer-hypoxia. The parametric calibration code and observational data used are available at: https://github.com/heberlr/Motility_Calibration. Image classifier was developed in Python3 using the OpenCV and NumPy libraries and is available at: https://github.com/heberlr/Image_Classifier. • This study did not generate/analyze data sets. • We used *xml2jupyter* (Heiland et al., 2019) to generate a Jupyter-based graphical user interface (GUI) from our PhysiCell model. An online interactive version of the model is available at: https://nanohub.org/tools/pc4tumorhypoxia.
