# Supplementary material for: A persistent invasive phenotype in post-hypoxic tumor cells is revealed by fate mapping and computational modeling
Source: iScience. 2021 Aug 4;24(9):102935. doi: 10.1016/j.isci.2021.102935 (PMC8449249; doi:10.1016/j.isci.2021.102935)
Supplement: Document S1. Figure S1–S9 and Table S1 [file mmc1.pdf]

iScience, Volume *24*

Supplemental information

A persistent invasive phenotype in post-hypoxic tumor cells is revealed by fate mapping and computational modeling

Heber L. Rocha, Inês Godet, Furkan Kurtoglu, John Metzcar, Kali Konstantinopoulos, Soumitra Bhoyar, Daniele M. Gilkes, and Paul Macklin

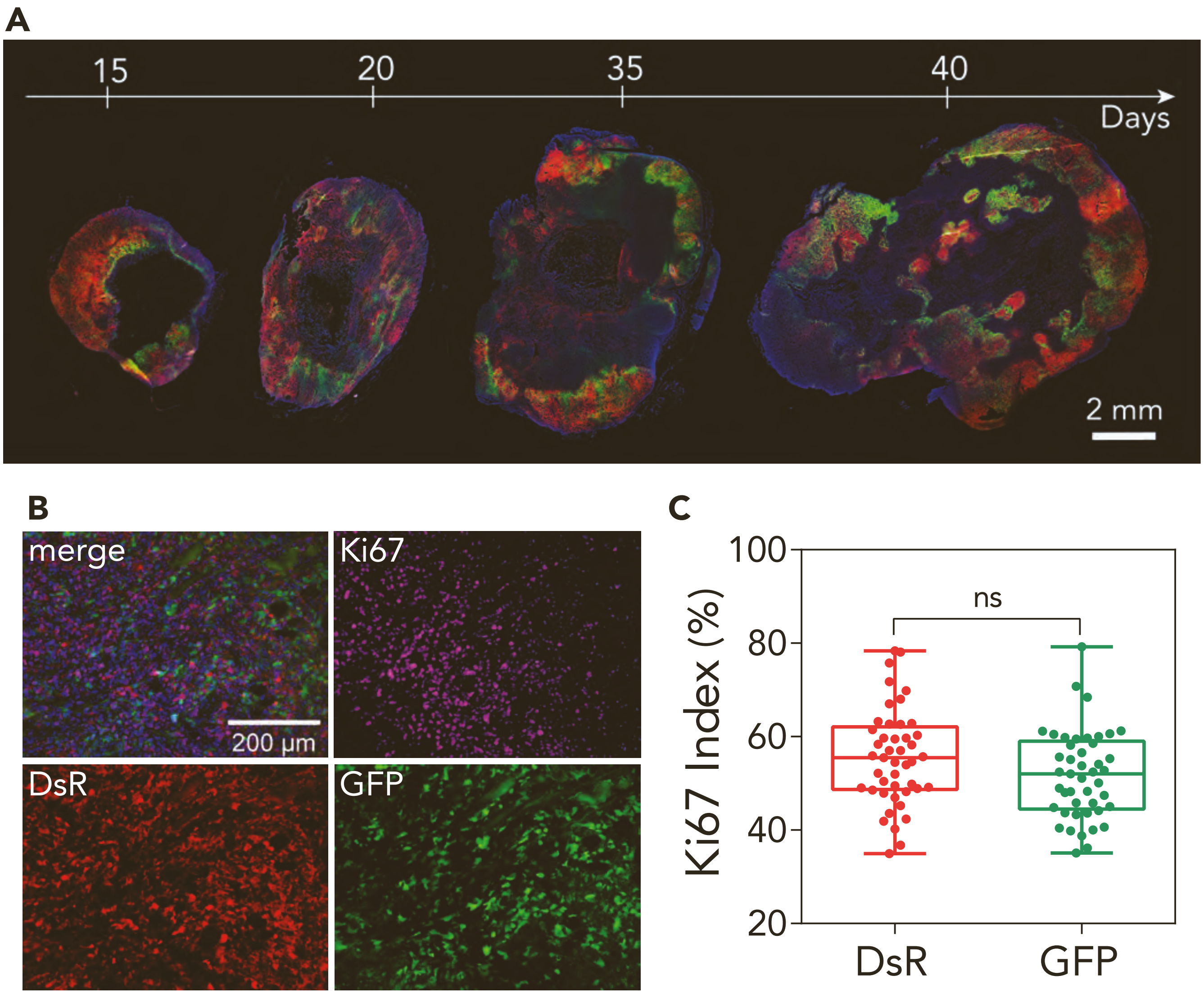

Figure S1. Fate-mapping intratumoral hypoxia and proliferation analysis, Related to Figure 2. (A) Fluorescent images of the full cross-sections of orthotopic tumors derived from MDA-MB-231 hypoxia fate-mapping cells. Tumors were excised at days 15, 20, 35 and 40 of the time course. (B-C) Representative fluorescent image (B) and quantification (C) of Ki67 staining in DsRed+ and GFP+ cells in a tumor section.

Parameter	Meaning	Value	Reference
U	oxygen consumption rate by cells	$10\ min^{-1}$	Ghaffarizadeh *et al.* 2016
D	oxygen diffusion coefficient	$10^5\ \mu m^2/min$	Ghaffarizadeh *et al.* 2016
λ	oxygen natural decay	$0.1\ min^{-1}$	Ghaffarizadeh *et al.* 2016
σ_0	initial oxygen pressure	$45.94\ mmHg$	Estimated
$\bar{\sigma}$	oxygen pressure at the edges of tumor	$45.94\ mmHg$	Godet *et al.* 2019
Initial_radius	radius of the initial tumor	$250\ \mu m$	Estimated
$\bar{r}_{01}$	trasition rate from Ki67- to Ki67+	$3.63 \times 10^{-3}\ min^{-1}$	Ghaffarizadeh *et al.* 2018
r_{10}	trasition rate from Ki67+ to Ki67-	$1.07 \times 10^{-3}\ min^{-1}$	Ghaffarizadeh *et al.* 2018
σ_S	oxygen pressure threshold to signal proliferation saturation	$38\ mmHg$	Estimated
σ_T	min oxygen pressure threshold to signal proliferation	$6\ mmHg$	Estimated
σ_H	oxygen pressure threshold to signal hypoxia	$10\ mmHg$	Godet *et al.* 2019
α_i	protein production rate	$4.8 \times 10^{-4}\ min^{-1}$	Estimated
β_i	protein degradation rate	$6.9 \times 10^{-5}\ min^{-1}$	Estimated

Table S1. Parameter values utilized in the simulations of the computational model, Related to Figure 4.

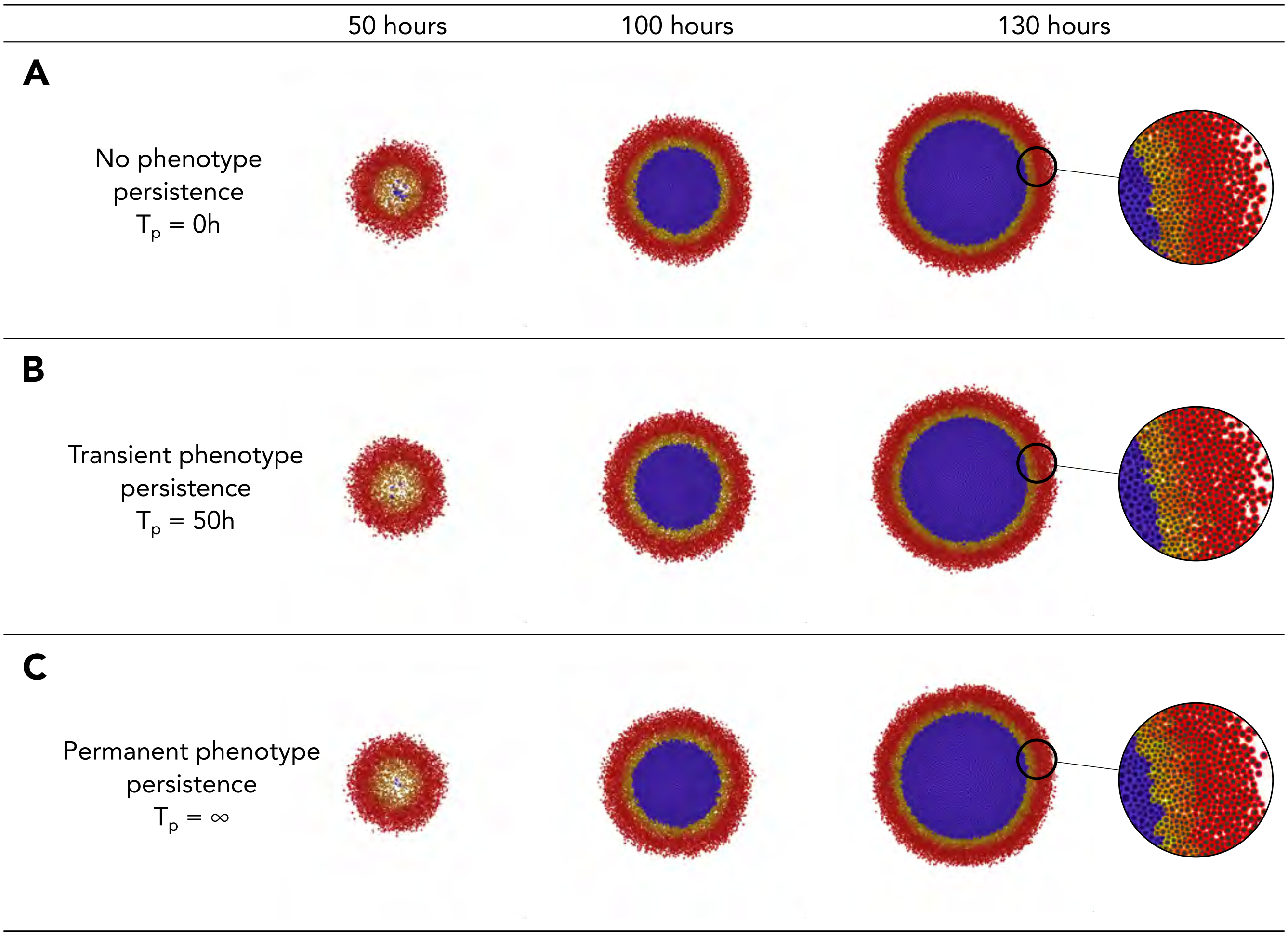

Figure S2. Impact of phenotypic persistence time with migratory bias fixed ($b = 0.1791$), Related to Figure 4. (A) No phenotypic persistence ($T_p = 0$). (B) Intermediate phenotypic persistence ($T_p = 50\ h$). (C) Permanent phenotypic persistence ($T_p = \infty$).

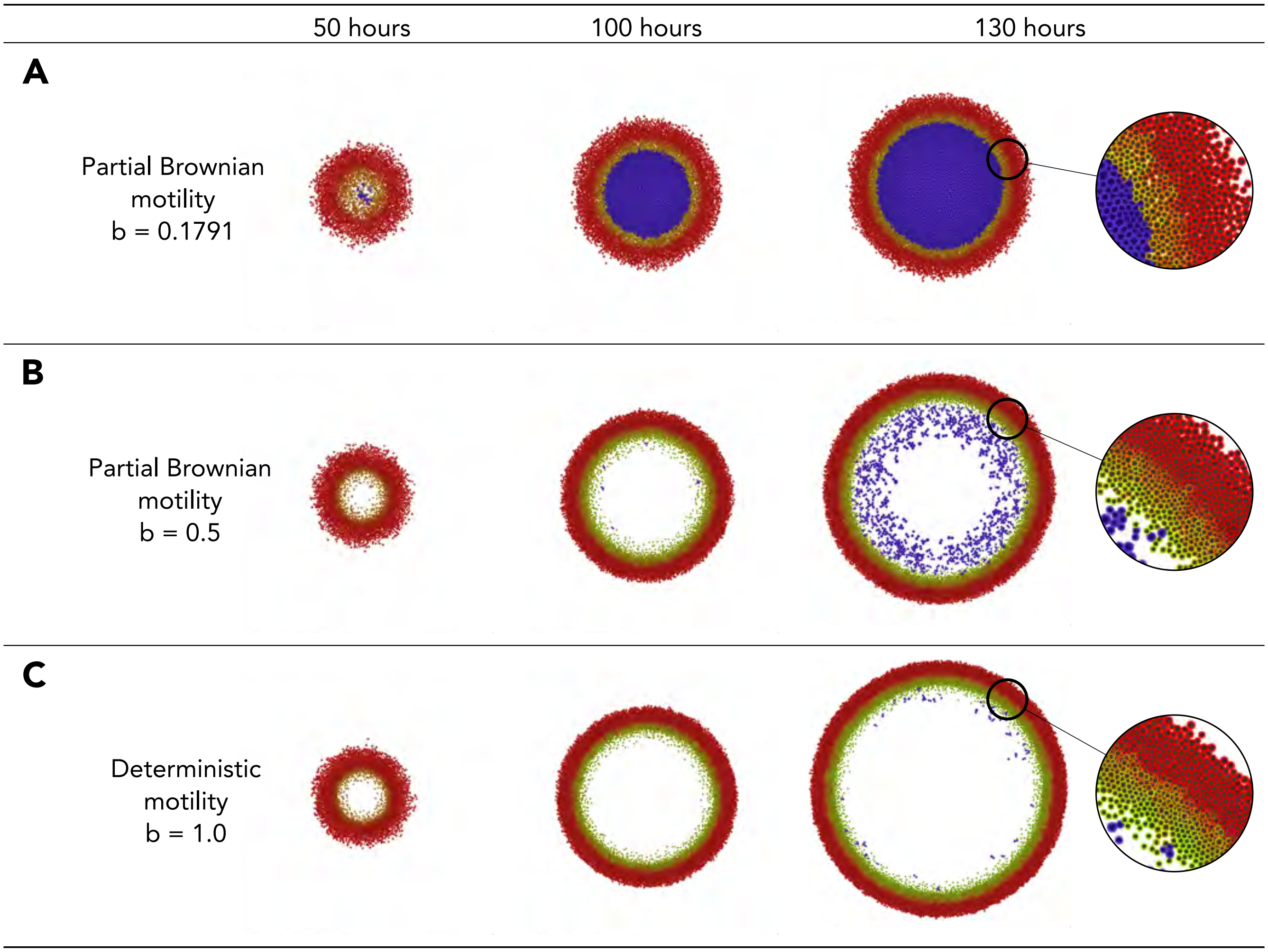

Figure S3. Impact of migratory bias without phenotypic persistence ($T_p = 0$), Related to Figure 4. (A) The motility bias generates a partial Brownian motility ($b = 0.1791$). (B) Intermediate motility bias achieves a more directed migration ($b = 0.5$). (C) Completely polarized movement ($b = 1.0$).

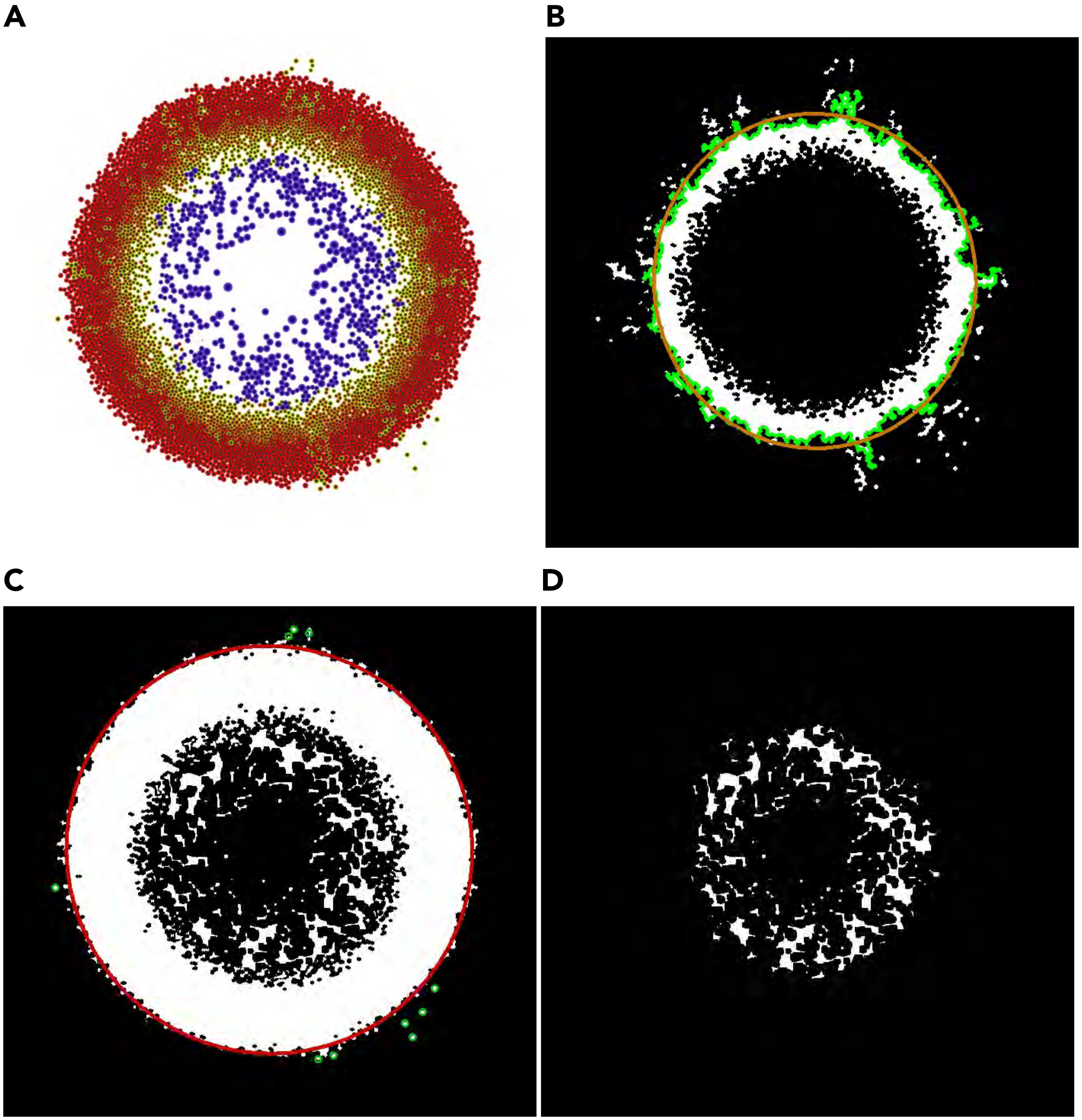

Figure S4. Image classifier output, Related to STAR Methods. (A) Output of the model using one simulation of the model at 100 hours with $F_r = 50\%$, $b^* = 0.5$, and $T_p = 50h$. (B) Image of the Boolean test for plumes. The function $\mathcal{F}_G$ in green and ellipse $\mathcal{E}_G$ in orange. (C) Image of the Boolean test for escaping cells. The ellipse $\mathcal{E}_T$ fitting the tumor in red, and the GFP+ cells escaping the tumor in green. (D) Image of necrotic area.

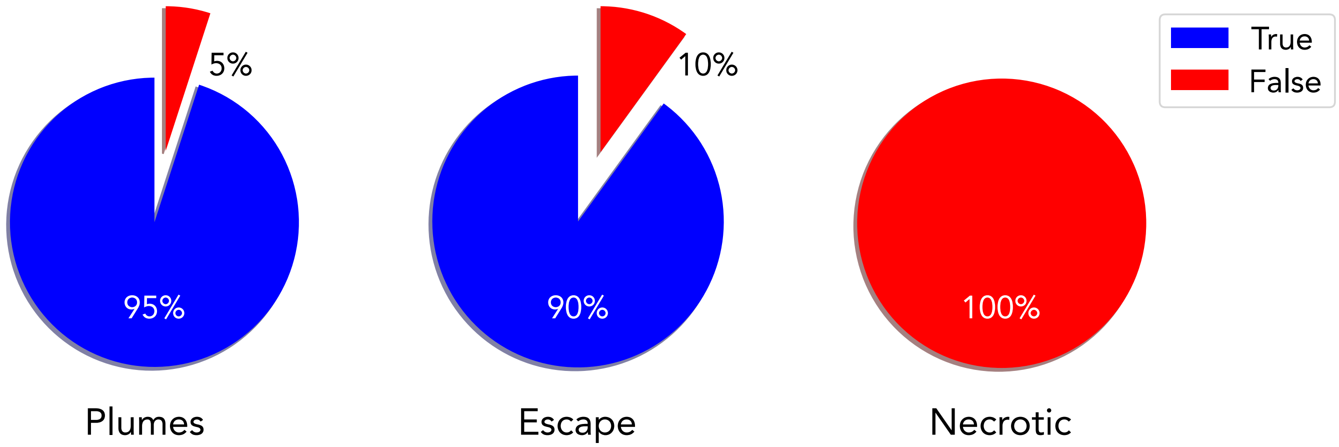

Figure S5. Boolean image classification, Related to STAR Methods. Percentage of the Boolean image classification of 20 replicates of the model (associated to 100h of tumor evolution), when $F_r = 50\%$, $b^* = 0.5$, and $T_p = 50h$.

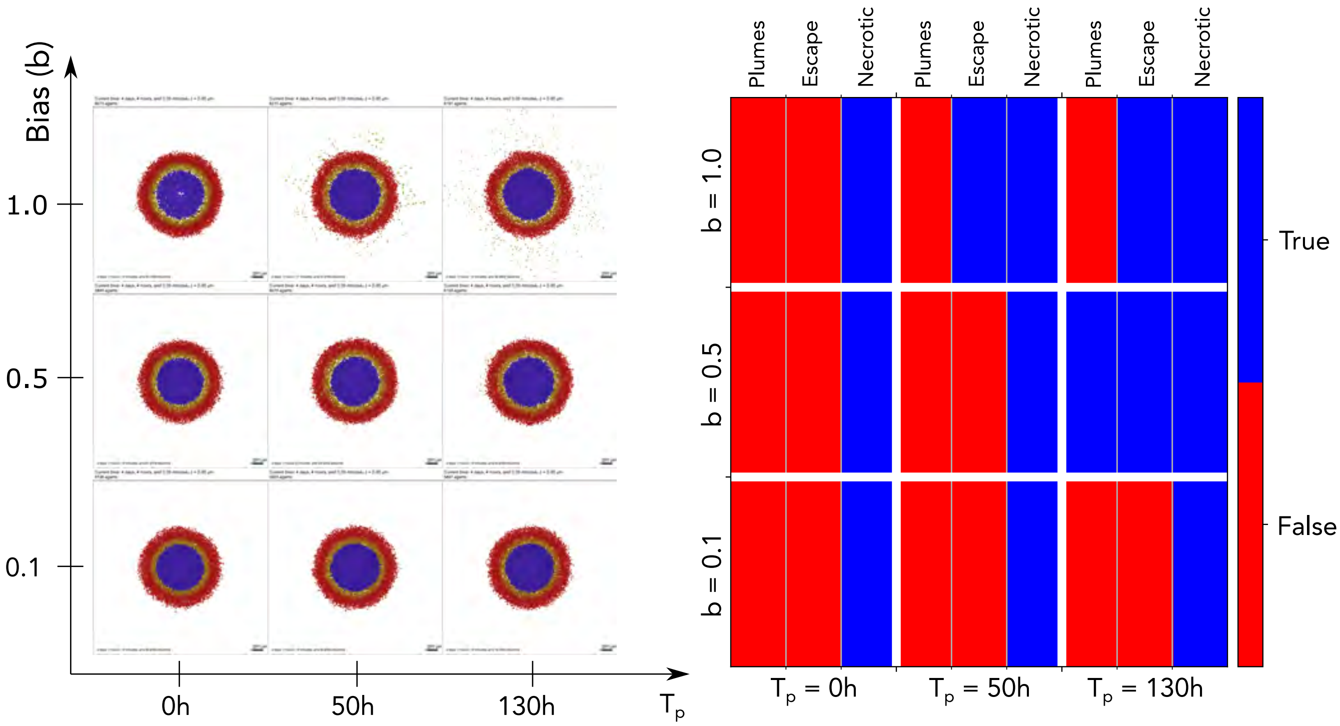

Figure S6. Study varying b and T_p with $F_r = 10\%$, Related to STAR Methods. Images resultant from the proposed model (left) and associated Boolean classification (right). All simulations were evaluated at 100 hours after the initial condition.

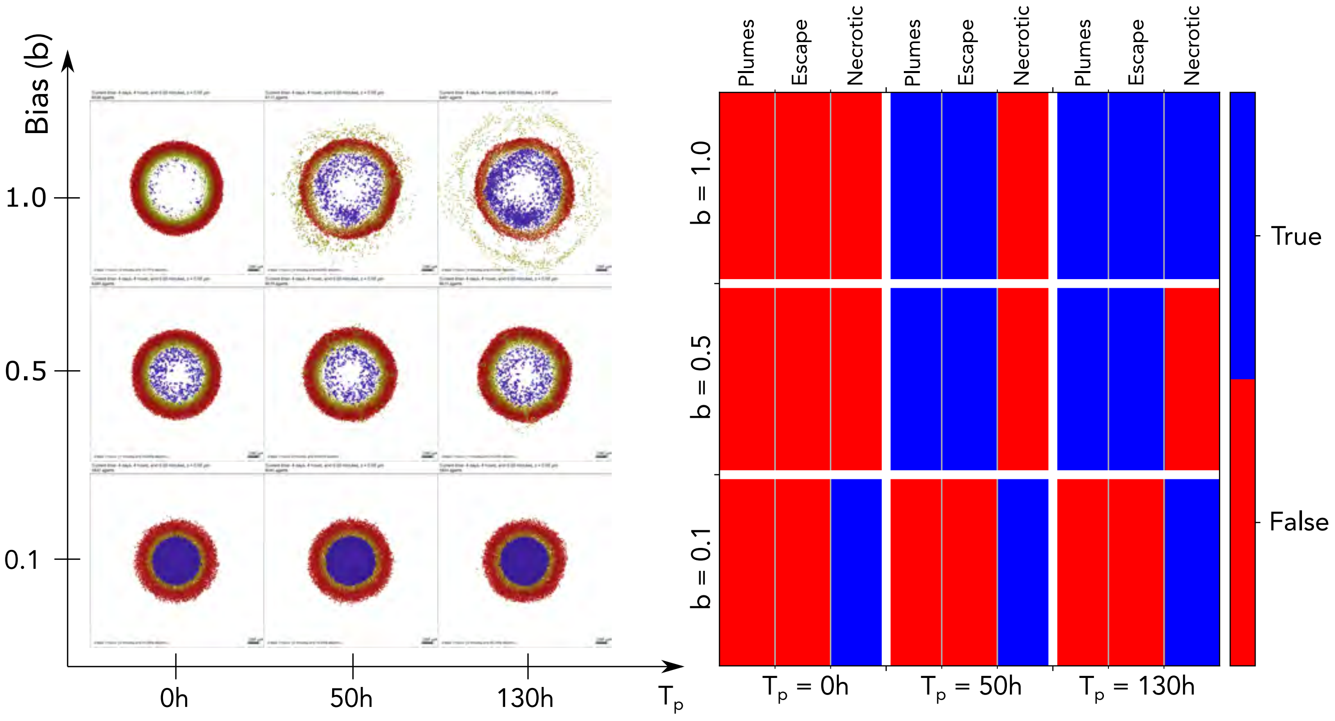

Figure S7. Study varying b and T_p with $F_r = 50\%$, Related to STAR Methods. Images resultant from the proposed model (left) and associated Boolean classification (right). All simulations were evaluated at 100 hours after the initial condition.

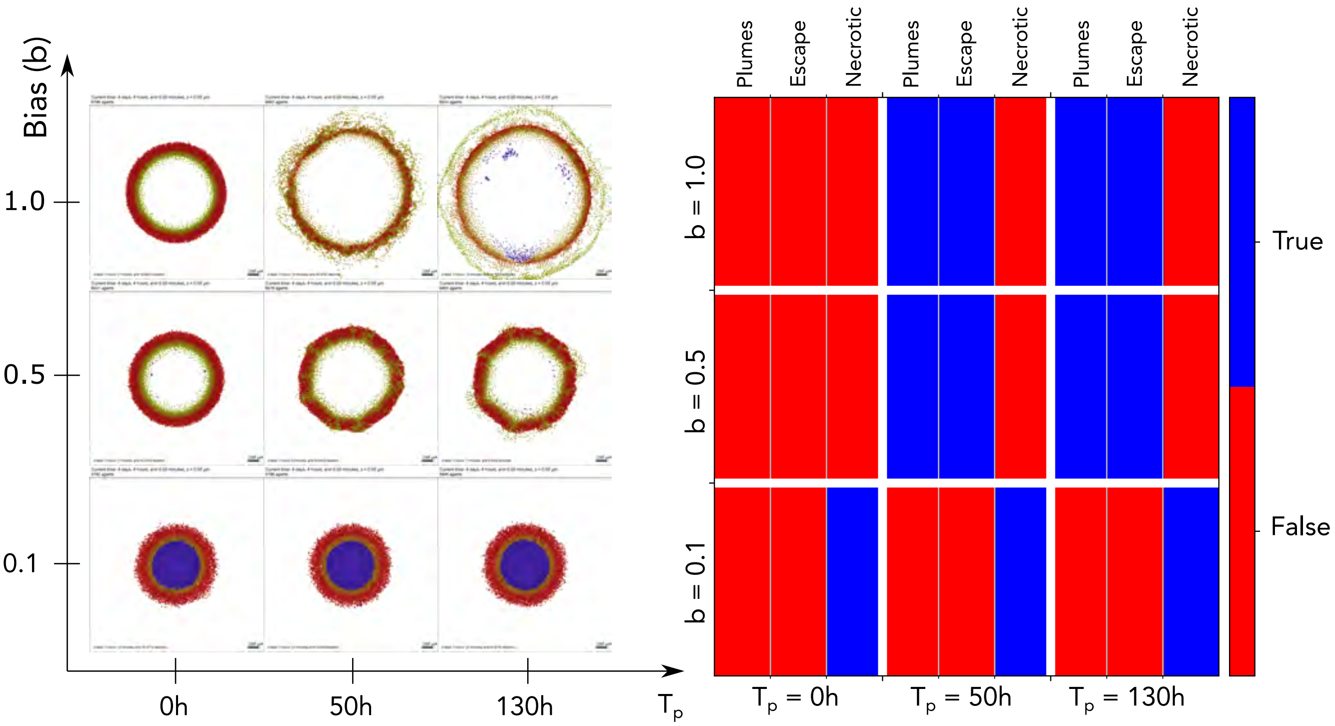

Figure S8. Study varying b and T_p with $F_r = 100\%$, Related to STAR Methods. Images resultant from the proposed model (left) and associated Boolean classification (right). All simulations were evaluated at 100 hours after the initial condition.

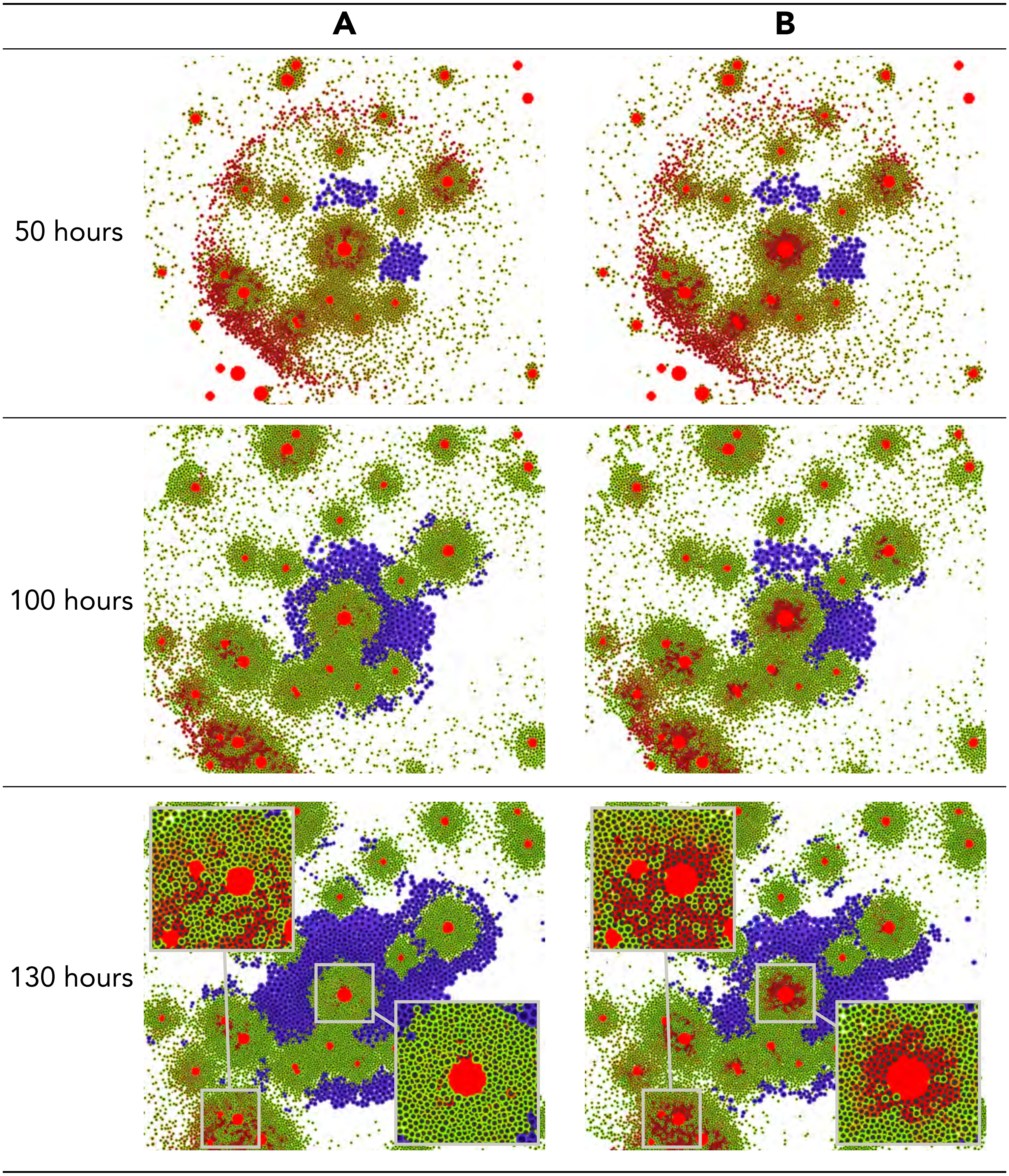

Figure S9. Impact of heterogeneous and multiple oxygen sources, Related to STAR Methods. (A-B) The simulation of the model for the case in which we arranged oxygen sources randomly in the computational domain, (A) adopting $F = 50$, $b = 0.5$, and $T_p = 50\ h$; and (B) adding mechanical feedback on proliferation and migration.
